# Bioethanol from poplar clone Imola: an environmentally viable alternative to fossil fuel?

**DOI:** 10.1186/s13068-015-0318-8

**Published:** 2015-09-04

**Authors:** Miao Guo, Changsheng Li, Gianni Facciotto, Sara Bergante, Rakesh Bhatia, Roberto Comolli, Chiara Ferré, Richard Murphy

**Affiliations:** Department of Chemical Engineering, Imperial College London, London, SW7 2AZ UK; Department of Life Sciences, Imperial College London, London, SW7 2AZ UK; Institute for the Study of Earth, Oceans, and Space, Morse Hall, University of New Hampshire, Durham, NH 03824 USA; Research Units for Intensive Wood Production (PLF), Agriculture Research Council (CRA), Casale Monferrato, Italy; Institute of Biological, Environmental and Rural Sciences, Aberystwyth University, Aberystwyth, Ceredigion SY23 3EB UK; Department of Environmental and Land Sciences, Milano Bicocca University, Milan, Italy; Centre for Environmental Strategy, University of Surrey, Guildford, Surrey, GU2 7XH UK

**Keywords:** 2G biofuel, Perenial bioenergy crop, Poplar, Bioethanol, Supply chain, Life cycle assessment, Carbon and nitrogen cycling, Biogeochemistry model, DNDC

## Abstract

**Background:**

Environmental issues, e.g. climate change, fossil resource depletion have triggered ambitious national/regional policies to develop biofuel and bioenergy roles within the overall energy portfolio to achieve decarbonising the global economy and increase energy security. With the 10 % binding target for the transport sector, the Renewable Energy Directive confirms the EU’s commitment to renewable transport fuels especially advanced biofuels. Imola is an elite poplar clone crossed from *Populus deltoides* Bartr. and *Populus nigra* L. by Research Units for Intensive Wood Production, Agriculture Research Council in Italy. This study examines its suitability for plantation cultivation under short or very short rotation coppice regimes as a potential lignocellulosic feedstock for the production of ethanol as a transport biofuel. A life cycle assessment (LCA) approach was used to model the cradle-to-gate environmental profile of Imola-derived biofuel benchmarked against conventional fossil gasoline. Specific attention was given to analysing the agroecosystem fluxes of carbon and nitrogen occurring in the cultivation of the Imola biomass in the biofuel life cycle using a process-oriented biogeochemistry model (DeNitrification-DeComposition) specifically modified for application to 2G perennial bioenergy crops and carbon and nitrogen cycling.

**Results:**

Our results demonstrate that carbon and nitrogen cycling in perennial crop–soil ecosystems such as this example can be expected to have significant effects on the overall environmental profiles of 2G biofuels. In particular, soil carbon accumulation in perennial biomass plantations is likely to be a significant component in the overall greenhouse gas balance of future biofuel and other biorefinery products and warrants ongoing research and data collection for LCA models. We conclude that bioethanol produced from Imola represents a promising alternative transport fuel offering some savings ranging from 35 to 100 % over petrol in global warming potential, ozone depletion and photochemical oxidation impact categories.

**Conclusions:**

Via comparative analyses for Imola-derived bioethanol across potential supply chains, we highlight priority issues for potential improvement in 2G biofuel profiling. Advanced clones of poplar such as Imola for 2G biofuel production in Italy as modelled here show potential to deliver an environmentally sustainable lignocellulosic biorefinery industry and accelerate advanced biofuel penetration in the transport sector.

**Electronic supplementary material:**

The online version of this article (doi:10.1186/s13068-015-0318-8) contains supplementary material, which is available to authorized users.

## Background

Transport is responsible for approximately 25 % of EU greenhouse gas (GHG) emissions and is the second largest sector for GHG emissions after energy [1]. More than two-thirds of transport-related GHG emissions are caused by road transport [2]. Dependency on imported fossil fuel has also increased over the last decades in the EU and nearly 84 % of the dominant transport fuel in the EU—fossil oil—is imported [3]. These issues have triggered ambitious national/regional policies to develop the role of biofuels and bioenergy within the overall energy portfolio of EU member states to achieve decarbonising the European economy and increase energy security. The EU 20/20/20 climate and energy targets set a 20 % share of renewable energy in final energy consumption by 2020 [4, 5]. With the 10 % binding target for the transport sector, the Renewable Energy Directive (RED) confirms the EU’s commitment to renewable transport fuels [6, 7]. Advanced biofuels derived from waste, agricultural or forestry residues, and lignocellulosic material will count twice towards this EU target [8]. Italy is playing a significant role in European burgeoning biofuel market. Following the success of launching world’s first commercial-scale advanced biofuel facility (Beta Renewables Ltd.) in Tortona in 2011, Italian government intends to extend its leading role by committing three new second generation (2G) biofuel plants [9]. Italy has a governmental action plan to increase the share of bio-resources in its energy mix [10] and is introducing a first national mandate for the application of advanced biofuels in the road transport sector, requiring 0.6 % of all petrol and diesel on the market to contain advanced biofuels from 2018, which increases to 1 % by 2022 [11].

Poplars (*Populus* spp.) have attracted significant interest for the potential in diverse applications including bioenergy and biofuel production due to its perennial habit, fast growth, ease of propagation, genetic diversity and range of traits [12, 13]. Poplar is a model hardwood species for breeding advanced genotypes due to its suitability for genetic manipulation with the availability of a complete genome sequence of *Populus trichocarpa* [14]. Research on poplar as a dedicated energy crop in Italy can be traced back to 1980s [10]. The present study focusses on *Populus* × *canadensis* Moench ‘Imola’, an Italian poplar elite clone obtained by controlled crossing of *Populus deltoides* Bartr. with *Populus nigra* L.

2G biofuels can be derived by various processing routes from lignocellulosic feedstocks, such as poplar. A major benefit of 2G biofuels is considered to be their potential to deliver very significant life cycle GHG emission reductions compared with fossil fuels (other benefits include minimisation of conflict with food crop production, capacity to use poorer marginal land, high yields of biomass per unit of land, diversity of potential feedstocks). Life cycle assessment (LCA) is a cradle-to-grave approach used to evaluate the environmental impacts of products and services. The LCA method has been formalised by the International Organization for Standardization in ISO 14040 series [15] and is becoming widely used to evaluate the holistic environmental aspects of various product systems and processes. The LCA framework, consisting of four phases: goal and scope definition, life cycle inventory analysis, impact assessment and interpretation. The guiding principles in conducting LCA are life cycle perspective, transparency and completeness, taking every environmental aspect and entire life cycle into account from raw material acquisition to final disposal. LCA methods have previously been applied to investigate the environmental footprint of 2G biofuels derived from bioenergy crops including poplar-derived bioethanol [16–20]. However, review of literatures indicates that such studies often lack precision or depth in accounting for linkages between the underlying biogeochemical processes and carbon and nitrogen cycles in perennial crop plantations and the overall environmental profiles generated for 2G biofuels. Although process-based models have been widely adopted for agricultural and forest ecosystems only very few studies have been carried out on the simulation of biogeochemical process underlying perennial bioenergy crop plantations [21–24]. With wide geographical scope, the process-based biogeochemistry model denitrification-decomposition (DNDC) appears to offer a potentially adaptable and applicable model to simulate C and N cycling for perennial energy crops based on its capacity to capture whole agro-ecosystem processes (including complex water and nutrient cycling) and to cover a wide range of crop types and regions [25]. DNDC was originally developed in 1992 for quantifying C sequestration and GHG emissions from US agricultural lands [26–29]. Over the past two decades, numerous updates have been implemented to DNDC to enhance its functionality and adapt it for various ecosystems and applications [30]. This study employed specific modifications to DNDC for perennial poplar plantations and applied the enhanced understanding of C/N cycling in evaluating the environmental profile of bioethanol production in Italy from the poplar clone Imola.

## Results and discussion

### DNDC simulation and Life Cycle Inventory (LCI) analysis for Imola plantation

The elemental analysis results for Imola in Table 1 provided the basis for developing the C/N partitioning regression model for DNDC. The field operations and the agrochemical and irrigation inputs modelled for SRC/VSRC Imola cycles over 10 years are given in Tables 2 and 3.

**Table 1:** Elemental analyses and enzymatic saccharification (mean ± standard deviation given in bracket)

	N element (% ODW)	C element (% ODW)	H element (% ODW)	Total glucose release (% ODW)
Stem-year 1	0.19 % (0.03)	49.61 % (0.39)	7.25 % (0.12)	6.70 % (0.32 %)
Stem-year 2	0.17 % (0.04)	49.42 % (0.17)	7.23 % (0.09)	10.95 % (0.64 %)
Stem-year 3	0.07 % (0.02)	49.56 % (0.18)	7.04 % (0.12)	7.79 % (0.39 %)
Branch-year 1	0.50 % (0.07)	50.96 % (1.12)	7.19 % (0.35)	7.96 % (0.28 %)
Branch-year 2	0.45 % (0.10)	49.26 % (0.53)	6.82 % (0.12)	10.16 % (0.54 %)
Branch-year 3	0.17 % (0.05)	49.64 % (0.64)	7.13 % (0.30)	8.00 % (1.27 %)
Leaf	2.49 % (0.16)	46.36 % (0.80)	6.48 % (0.10)	–
Corse root	1.07 % (0.08)	47.78 % (0.20)	6.42 % (0.11)	–
Fine root	1.38 % (0.09)	48.23 % (0.22)	6.57 % (0.13)	–

**Table 2:**
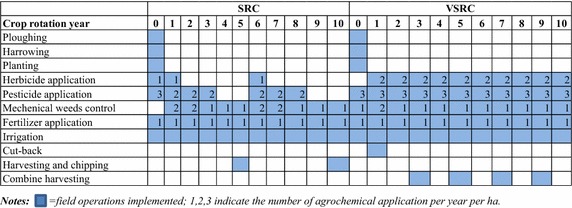
Crop regime planning—field operations for SRC/VSRC

**Table 3:** Inventory for agrochemical inputs and field operations per ODW tonne of Imola poplar harvested

	Application rate (kg agrochemical or m^3^ water)		Agricultural machinery		Energy inputs (MJ diesel for field operations or MJ electricity for irrigation)^c^	
	VSRC	SRC	VSRC	SRC	VSRC	SRC
Ploughing	NA	NA	Tractor (95 kW), three-furrow plough		1.05E+01	1.12E+01
Harrowing	NA	NA	Tractor (70 kW), harrow		5.04E+00	5.36E+00
Planting	NA	NA	Tractor (60 kW)		2.29E+00	9.75E+00
			Drill	Rotary machine		
Mechanical weed control	NA	NA	Tractor (44 kW), rotary machine		3.11E+01	3.40E+01
Herbicide	3.52E−01	9.39E−02	Tractor (60 kW), sprinkling machine		1.30E+01	9.51E+00
Pesticide	1.31E−01	3.83E−02	Tractor (70 kW), sprinkling machine			
N fertilizer	3.33	3.06	Tractor, spraying machine assumed for mechanical application^a^		8.02E+00	7.37E+00
Irrigation system installation	NA	NA	Excavator (12.41 kW)		6.69E−01	5.53E−01
Irrigation	2.37E+02	1.86E+02	Pump^b^		2.60E+02	2.14E+02
Harvesting	NA	NA	Harvester 250 kW	8.25E+01	8.25E+01	1.05E+02

*NA* Not applicable

^a^Fertilizers are manually apply in trails

^b^Electricity is used for irrigation currently in trial, but an improved energy efficiency for irrigation could be expected at commercial scale

^C^The density and low heating value of diesel assumed as 0.83 kg/L and 43.4 MJ/kg, respectively

Generally, Imola plantation represents an energy-efficient agricultural system compared with literature data [31]. Less energy and fewer agrochemical inputs were needed per unit harvested biomass for the SRC than for the VSRC regime due to higher SRC biomass yield (Table 3). Amongst field operations, irrigation was the dominant energy demand requiring approximately 63 and 54 % of total energy consumed for VSRC and SRC field operations, respectively (Table 3). The energy consumption for irrigation in the current study (1.12–1.15 MJ/m^3^) is within the range (0.13–7.7 MJ/m^3^) reviewed by Nonhebel [32] and somewhat lower than energy inputs reported by Mantineo et al. [33] and Sevigne et al. [31] (4.8 MJ/m^3^ in Italy and 3.1–3.2 MJ/m^3^ in Spain, respectively).

**Fig. 1:**
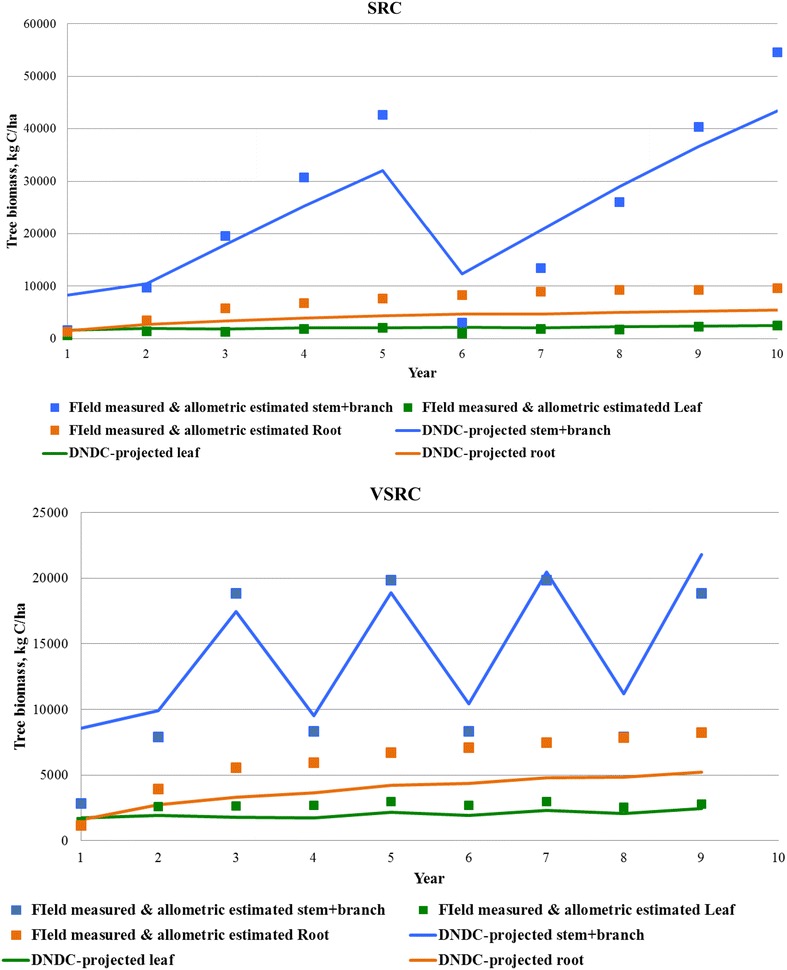
DNDC-simulated C pool vs. field measurements

To test applicability of the newly modified DNDC model to the Imola poplar perennial bioenergy crop, DNDC-simulated results were compared with field measurements. Biomass yields and C partitioning between stem (plus branch), leaves and roots derived from the DNDC simulations showed good agreement with the experimental observations (Fig. 1).

**Fig. 2:**
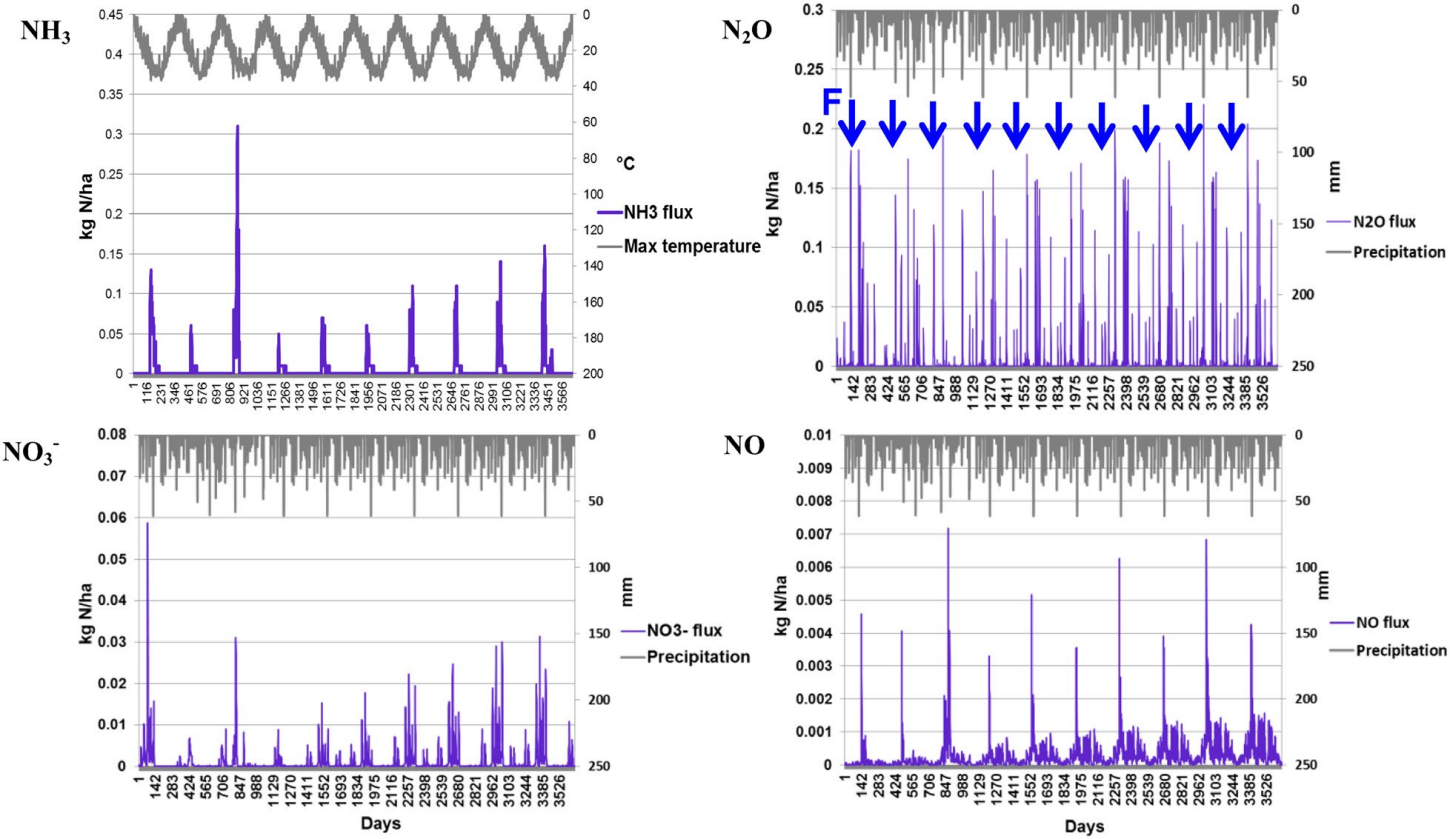
DNDC-projected daily N fluxes over 10-year VSRC Imola poplar cycles (2009–2019). *Days* Julian days. *Arrows* fertilizer inputs

**Fig. 3:**
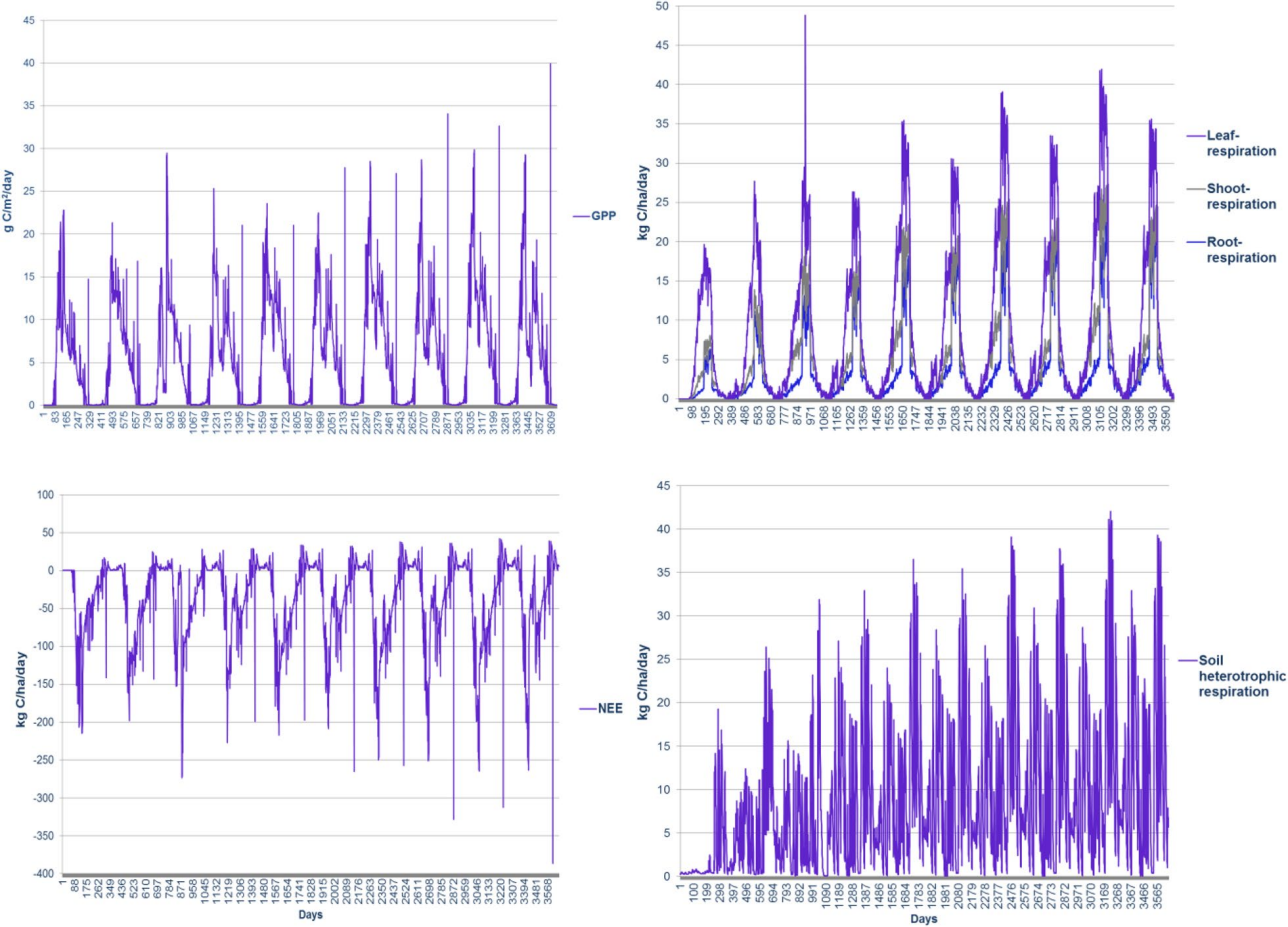
DNDC-projected daily C fluxes over 10-year VSRC Imola cycles (2009–2019). *Days* Julian days. C fluxes derived from DNDC simulation include gross primary production (GPP), plant respiration (leaf/shoot/root respiration), net ecosystem exchange of carbon (NEE) and soil heterotrophic respiration

As presented in Table 4, DNDC-simulated total soil N loss due to plant uptake, nitrate leaching and N gas emissions was about 8.2 kg N per oven dry weight (ODW) ton of Imola harvested, among which plant uptake accounts for 94 %, N field emissions in total contribute 6 %. VSRC and SRC Imola show similar N flux patterns (Table 4)—DNDC-simulated N emissions over 10-year rotation are dominated by N_2_O (30–36 %), NH_3_ (24–26 %) and N leaching (21–24 %), which imply low soil buffering effects (sandy texture soil with low organic matter and clay contents). As presented in Fig. 2, DNDC-simulated daily N_2_O emission peaks and N leaching are strongly related to N fertilizer inputs and rainfall events, which trigger the anaerobic zones developed in the soil. NH_3_ emission (by volatilisation) peaks roughly match the daily maximum temperature trends. The DNDC-simulated daily carbon fluxes are shown in Fig. 3. Gross primary production (GPP) describes the rate, at which the plant produces useful chemical energy and is defined as the total amount of carbon fixed by photosynthesis [34]; whereas, net ecosystem exchange (NEE) of carbon is equivalent to the difference between GPP and ecosystem respiration (ER) [35, 36]. ER is the biotic conversion of organic carbon to carbon dioxide by all organisms in an ecosystem [37] accounting for plant respiration (root, shoot and leaf) and microbial heterotrophic respiration. In DNDC, plant respiration is simulated at a daily time step by considering the effects of environmental drivers, e.g. atmospheric temperature, N availability; whereas, microbial heterotrophic respiration is calculated by simulating soil organic carbon decomposition in DNDC [36]. The methane flux is predicted by modelling CH_4_ production, oxidation and transport process [36]. As shown in Table 4 and Fig. 3, DNDC projected negative NEE and CH_4_ oxidation fluxes, which indicated a net uptake of CO_2_ by the plant–soil ecosystem and a net CH_4_ sequestration by oxidation process, respectively. DNDC-simulated NEE values (11.5–15.4 ton C/ha/year) in current study are higher than measured NEE (0.96–9.6 ton C/ha/year) reported for poplar plantation in previous research [38–40]. A strong link of annual NEE with stress conditions or extreme climate (e.g. high temperature) was suggested based on previous empirical work [40], a higher annual NEE could be expected in regions characteristic of high temperature like Italy. The simulated NEE for SRC and VSRC plantation is 0.688 and 0.768 ton C/ton ODW harvested Imola, respectively. Based on NEE and carbon sequestered in Imola biomass [about 50 % of ODW biomass (Table 1)], soil carbon sequestration is calculated as 0.19 ton C/ton harvested DOW SRC and 0.26 ton C/ton harvested DOW VSRC biomass, which is higher than the data range in previous studies (6–24 % of the total above-ground woody biomass) [41–46]. However, research on forest/plantation and associated soil carbon sink still remain scarce [47], some potential carbon pools under SRC/VSRC plantation might have been overlooked, e.g. weed root C inputs to soil [48]. To further validate the applicability of modified DNDC to perennial crops and advance the understanding of C and N cycling in Imola crop–soil ecosystems, comparisons of DNDC simulation with measurement obtained from eddy covariance system would be needed in future research.

**Table 4:** DNDC-simulated C/N fluxes over 10-year rotation (per ODW tonne Imola poplar harvested)

C/N flux	SRC	VSRC
GPP (kg C/ton)	−1.09E+03	−1.11E+03
Plant respiration (kg C/ton)	2.19E+02	1.76E+02
Soil heterotrophic respiration (kg C/ton)	2.02E+02	1.65E+02
NEE (kg C/ton)	−6.88E+02	−7.68E+02
CH_4_ oxidation (kg C/ton)	−9.29E−02	−1.00E−01
N uptake (kg N/ton)	7.51E+00	7.86E+00
N leaching (kg N/ton)	1.09E−01	1.21E−01
N Runoff (kg N/ton)	1.53E−03	1.73E−03
N_2_O (kg N/ton)	1.82E−01	1.53E−01
NO (kg N/ton)	6.28E−03	5.51E−03
N_2_ (kg N/ton)	8.63E−02	9.42E−02
NH_3_(kg N/ton)	1.24E−01	1.30E−01

### LCI for bioethanol production

The results in Table 1 show that, without pretreatment, total glucose yields from enzymatic saccharification vary with age of harvested Imola (within the range of 6–10 % of ODW). One-way analysis of variance (ANOVA) tests indicated no statistically significant variation between glucose releases from Imola stem and branch in each age group. Thus in this study, a simplified assumption was adopted that Imola stem and branch (with bark) were processed together at the biorefinery for bioethanol production. However, in future research further laboratory experiments will be needed (e.g. compositional analyses, pretreatment, saccharification) to investigate the processability of Imola components (stem/branch) before and after different pretreatment technologies and to generate a site-specific LCA inventory for Imola-derived bioethanol production.

Based on biorefinery simulation model, a summarised inventory for biorefinery is given in Additional file 1: Table S1. The transport involved in the Imola-derived bioethanol supply chains is derived from [19] and given in Additional file 1: Table S2. These include on-site transport, i.e. transport of harvested Imola wood from field to plantation gate, transport of biomass to biorefinery and transport of bioethanol from biorefinery to forecourt.

Biorefinery model data along with the DNDC simulation and agricultural inputs were used as LCA inventory.

### Cradle-to-farm-gate life cycle impact assessment (LCIA) profiles for Imola biomass feedstock production

**Fig. 4:**
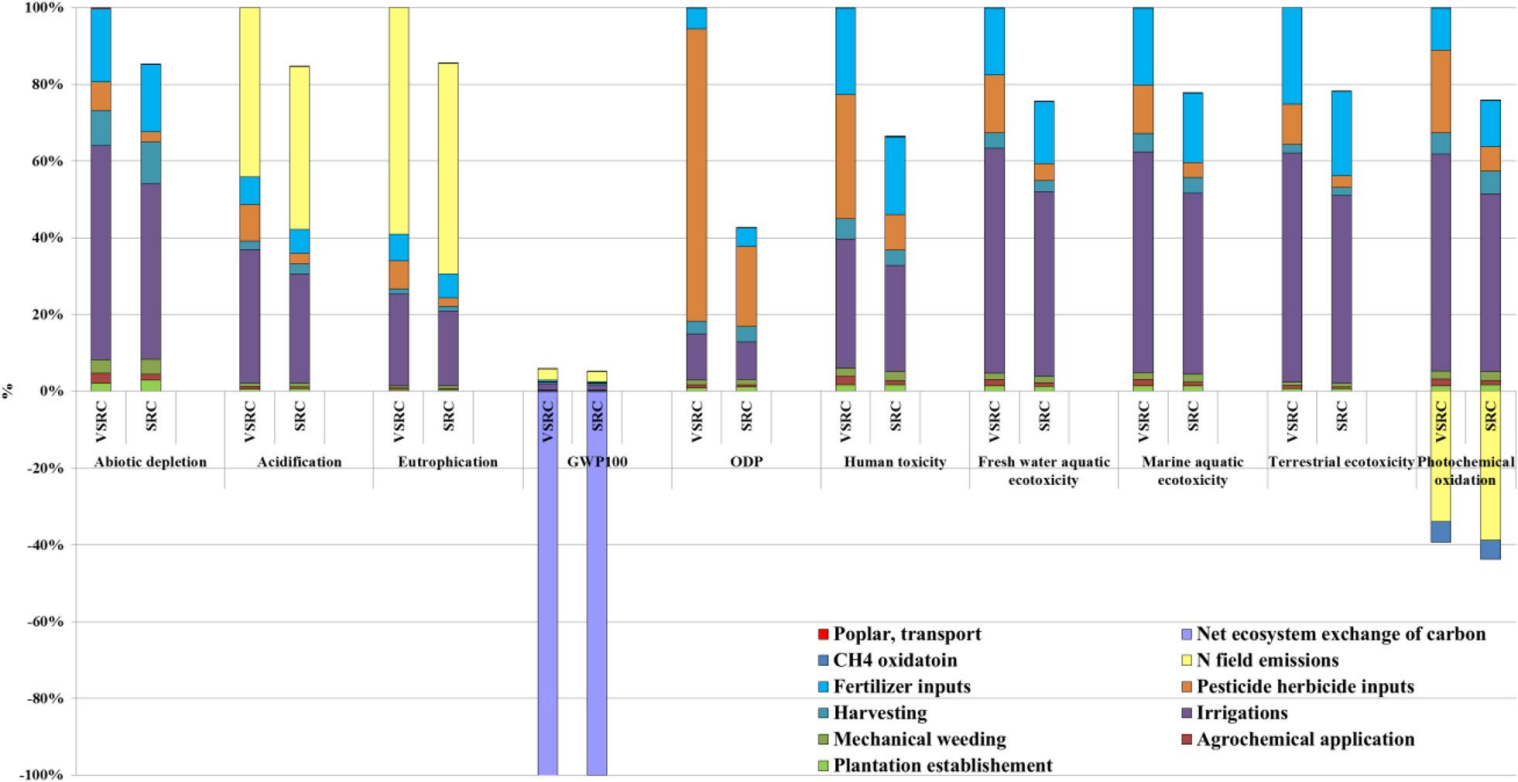
Characterised LCIA profiles of Imola biomass at farm gate per kg ODW Imola poplar biomass. LCIA characterisation method: CML 2 baseline 2000

**Fig. 5:**
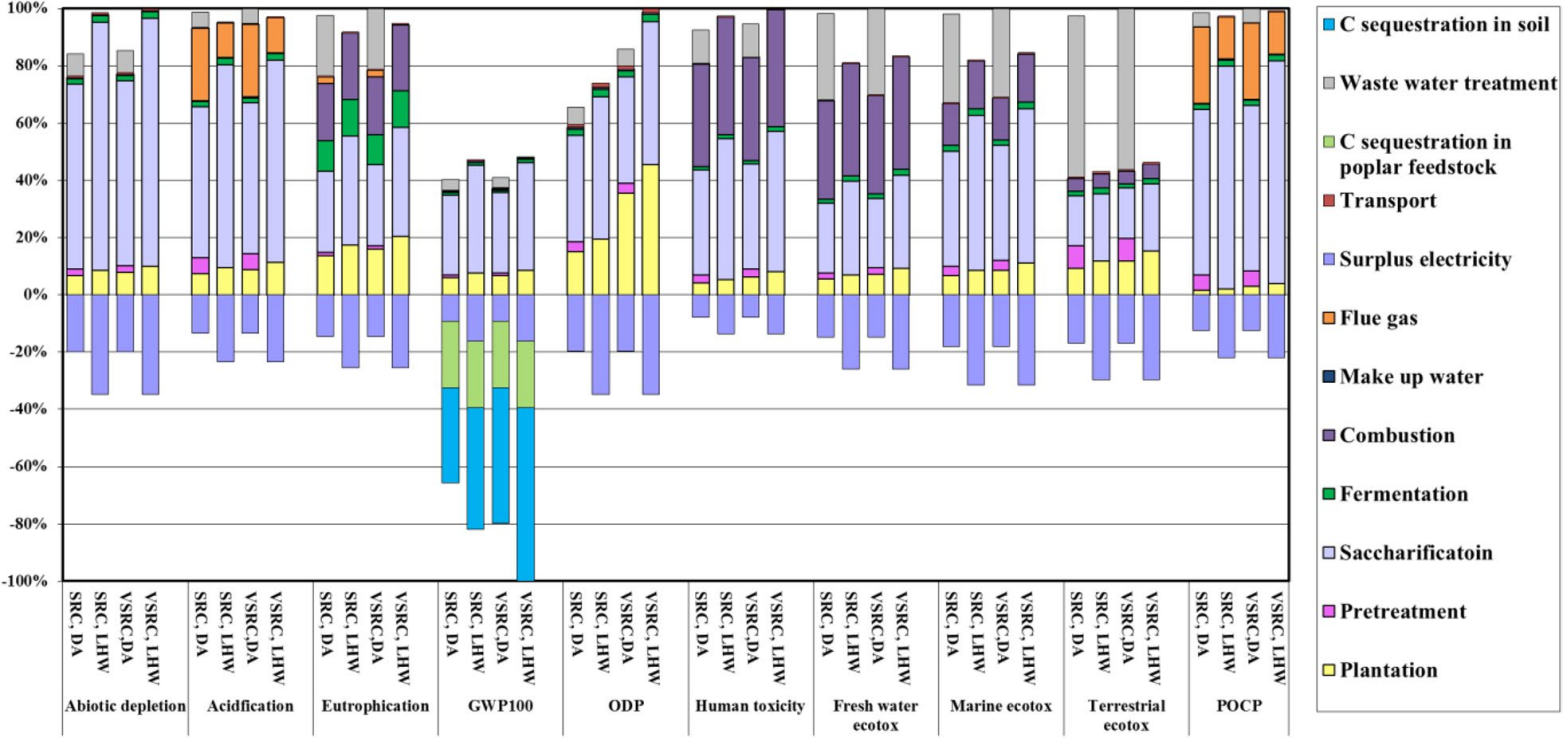
Characterised LCIA profiles of Imola-derived bioethanol at biorefinery gate per kg bioethanol. LCIA characterisation method: CML 2 baseline 2000

**Fig. 6:**
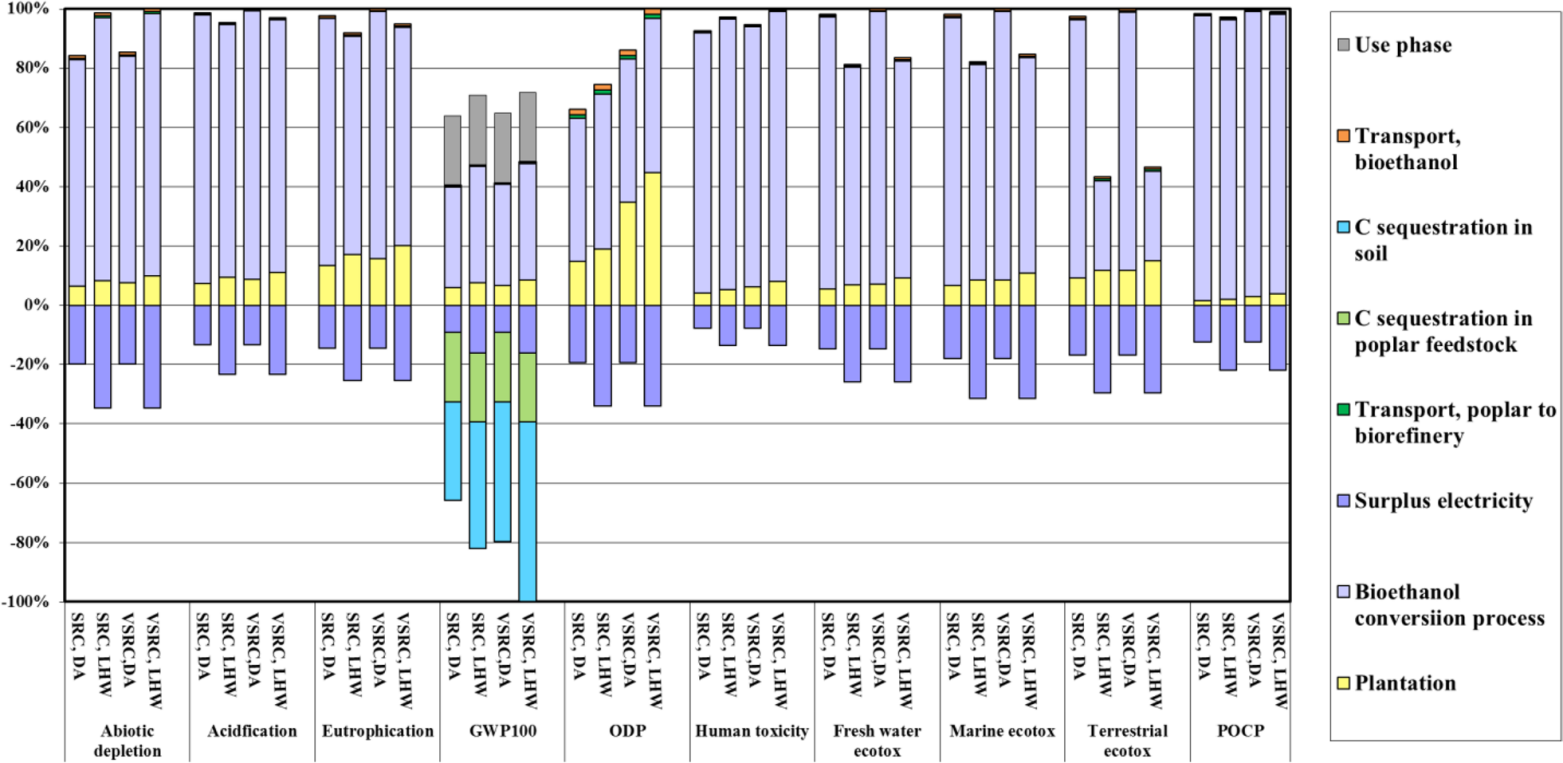
Characterised LCIA comparison of E100 bioethanol over life cycle per functional unit. Functional unit: 100 km driven in a FFV. LCIA characterisation method: CML 2 baseline 2000

**Fig. 7:**
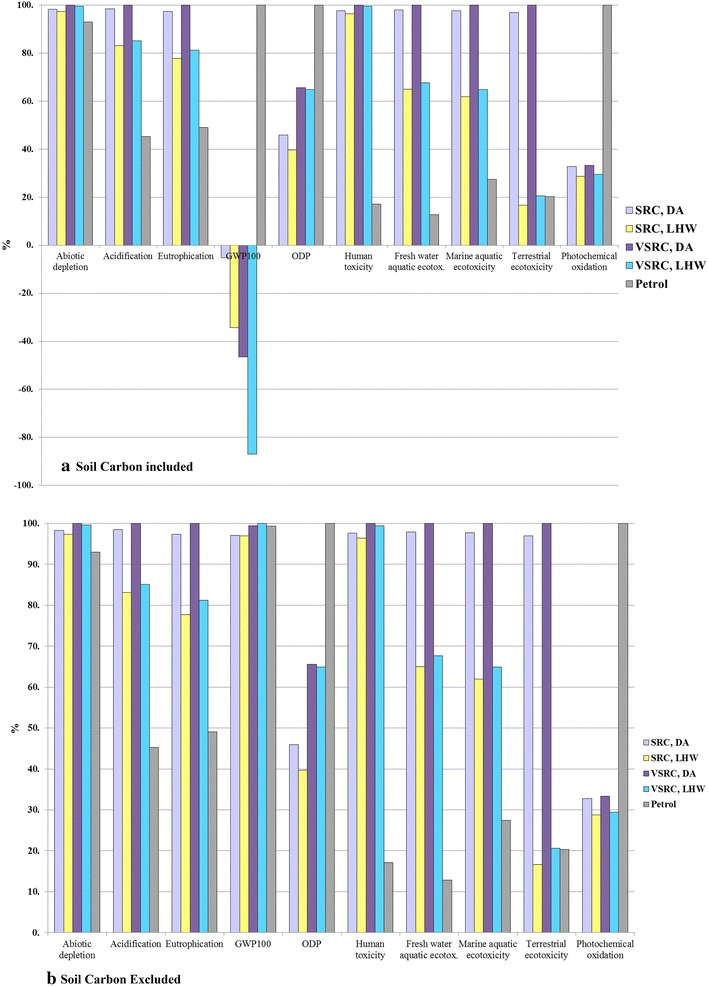
Characterised LCIA comparison of E100 bioethanol vs. petrol per functional unit. Functional unit: 100 km driven in a FFV. LCIA characterisation method: CML 2 baseline 2000

**Fig. 8:**
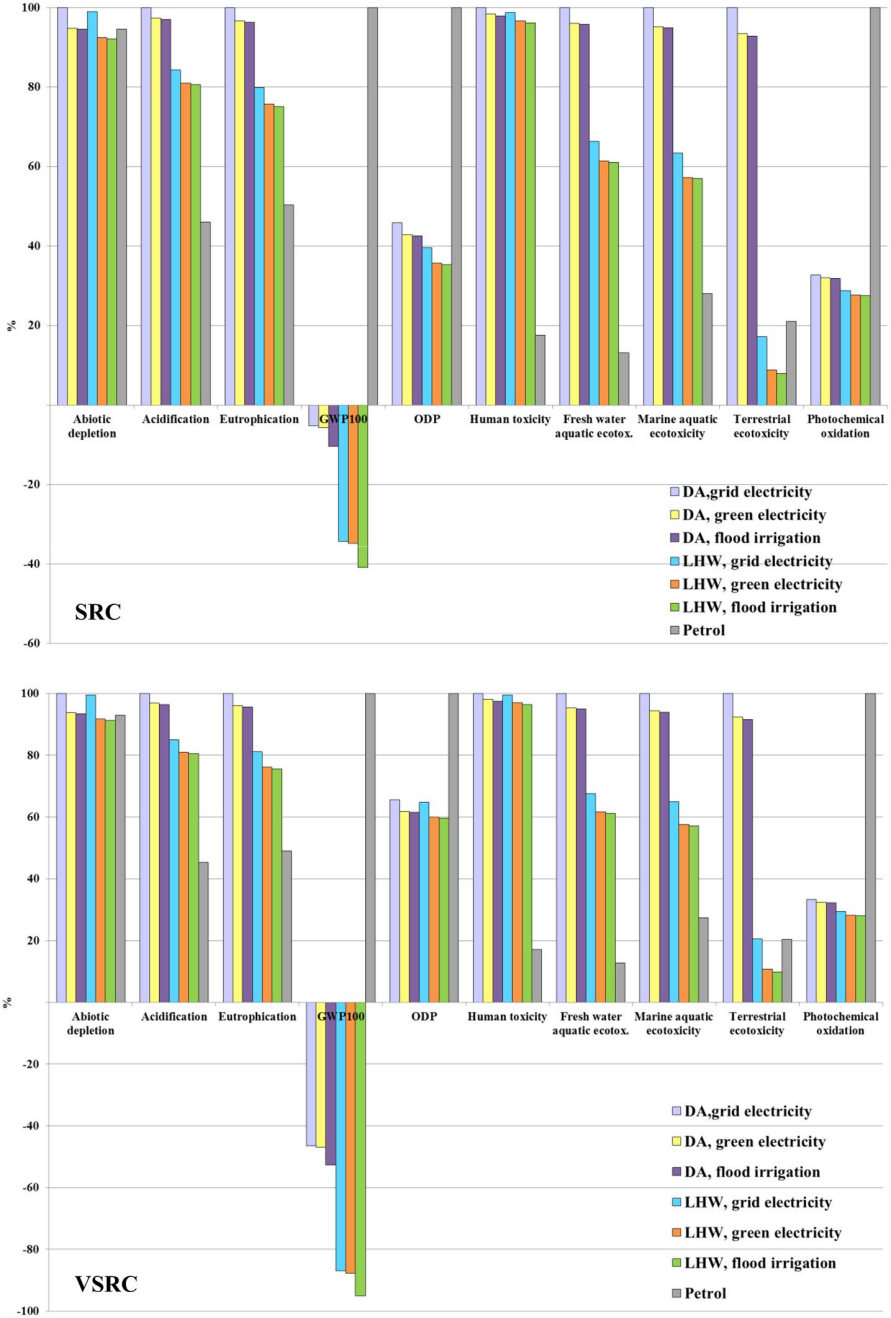
Characterised cradle-to-grave LCIA comparison of E100 bioethanol from SRC and VSRC Imola per functional unit. Functional unit: 100 km driven in a FFV. LCIA characterisation method: CML 2 baseline 2000

**Fig. 9:**
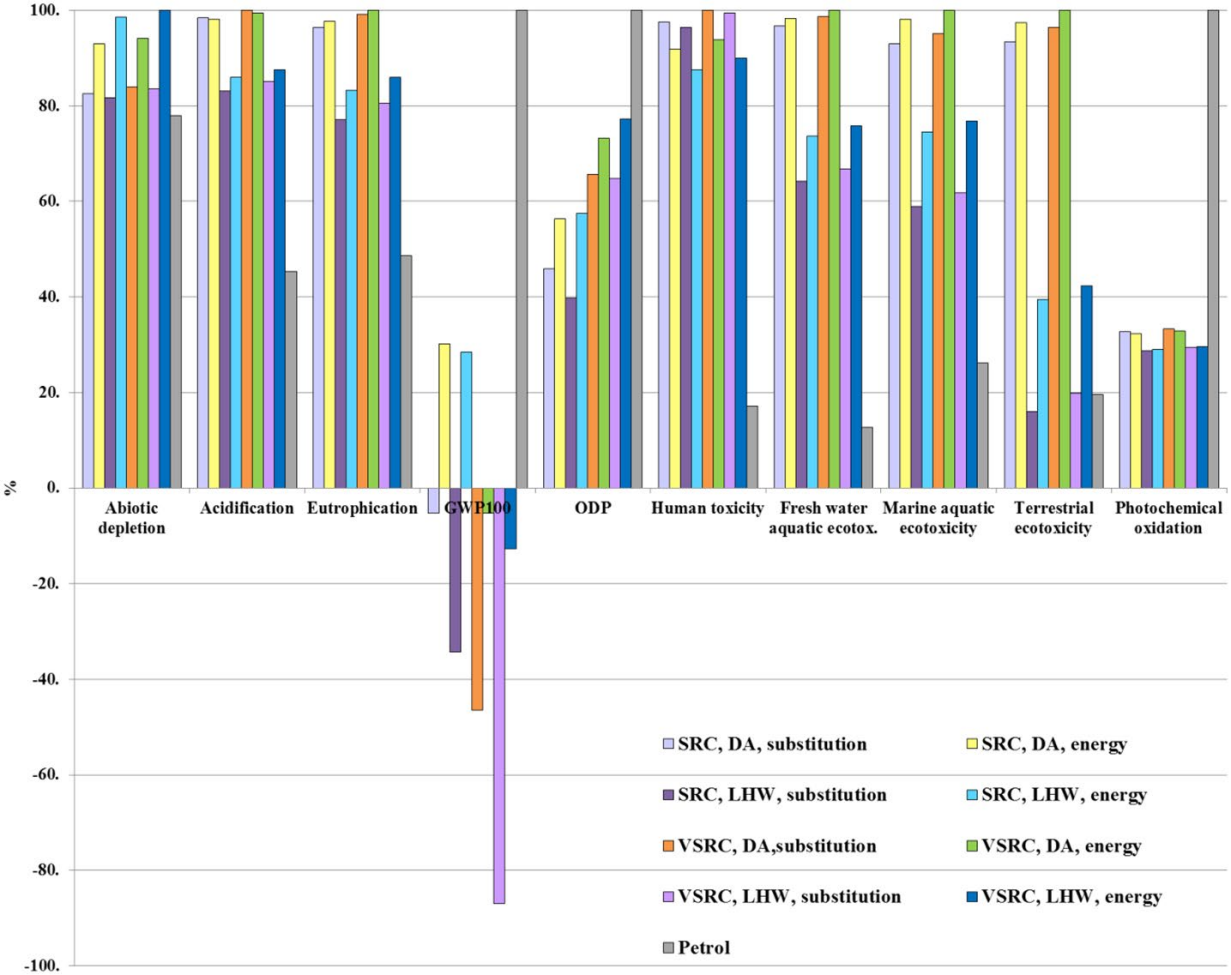
Sensitivity analyses on allocation approach per functional unit. Functional unit: 100 km driven in a FFV. LCIA characterisation method: CML 2 baseline 2000

**Fig. 10:**
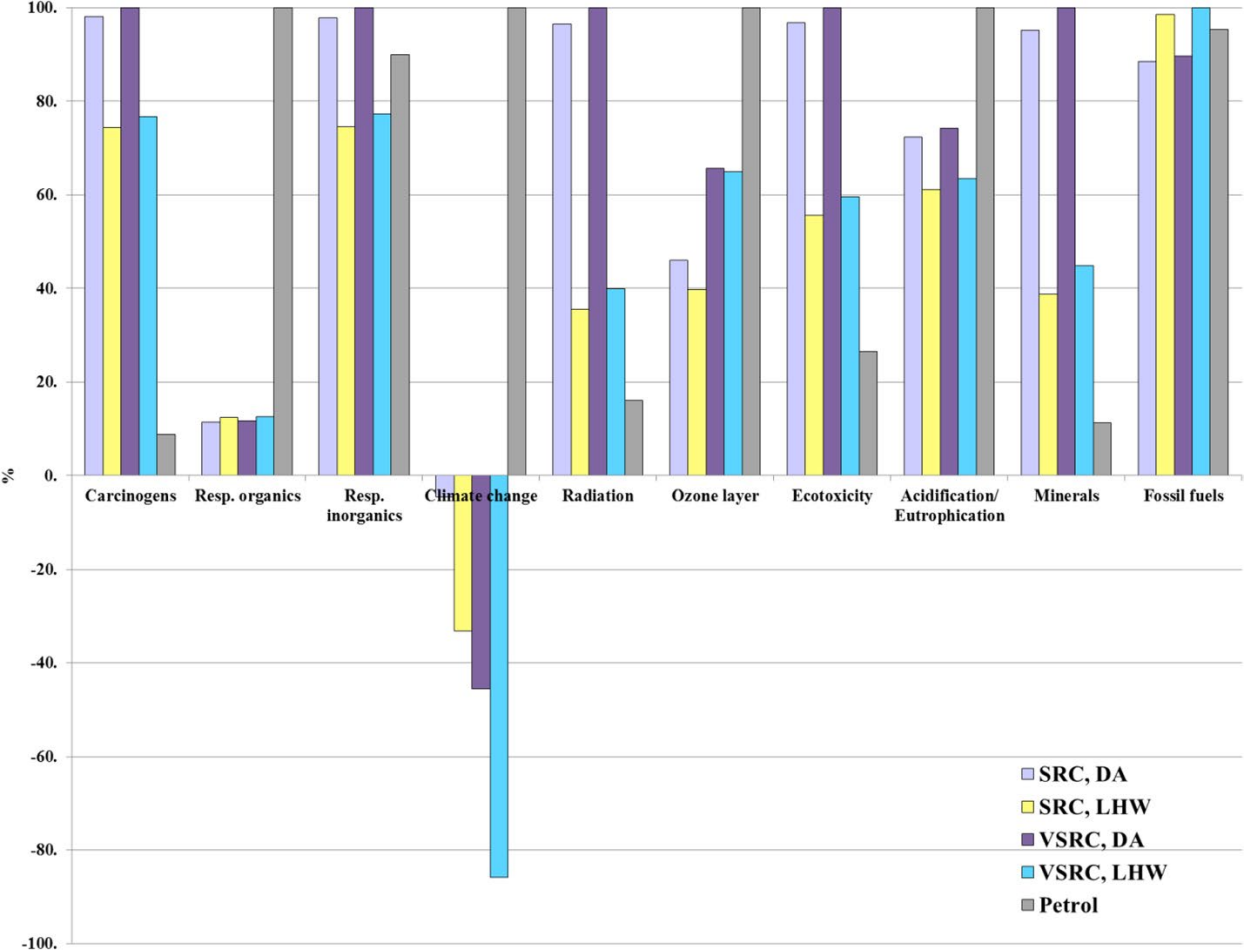
Sensitivity analyses on characterisation models per functional unit. Functional unit: 100 km driven in a FFV. LCIA characterisation method: Eco-indicator 99 Hierarchist

The results for all LCIA have been presented as normalised comparisons (%) in Figs. 4, 5, 6, 7, 8, 9 and 10. The LCIA scores for each individual impact category and scenario are given in Additional file 1: Tables S3-S16. Overall, irrigation, agrochemical inputs and the induced field emissions are the dominant factors driving the cradle-to-farm-gate environmental profiles of Imola biomass cultivated under different plantation management regimes, i.e. SRC and VSRC (Fig. 4). Similar profiles are found on abiotic depletion, human and eco-toxicities impact categories, where irrigation and agrochemical inputs (fertilizers, pesticide and herbicides) cause 40–60 and 25–55 % of the environmental impacts, respectively. These impacts are due to the demand for grid electricity (for irrigation) from natural gas, fuel oil and coal in Italy (i.e. fossil resource consumption and toxicants, e.g. nickel beryllium, chromium, vanadium emitted during fossil fuel extraction and combustion) and the energy-intensive production processes for pesticides, herbicides and N fertilizers. Additionally, electricity for irrigation contributes 20–40 % environmental burdens on acidification, eutrophication, global warming potential (GWP_100_) and approximately 60 % of positive impacts on photochemical oxidation (POCP) due to the atmospheric emissions (NH_3_, SO_x_, NO_x_, CH_4_ and CO) released from natural gas, fuel oil and coal combustion and phosphorus emitted to water during coal production. 50–60 % of environmental burdens on acidification, eutrophication and GWP_100_ are attributed to N field emissions simulated in the agro-ecosystem DNDC modelling including NH_3_, N_2_O, NO and N leaching, whereas NO combined with net CH_4_ sequestration led to beneficial POCP effects (presented as negative scores below line). Such negative POCP scores are mainly attributable to the removal of CH_4_ from atmosphere by oxidation processes and removal of O_3_ via the atmospheric reaction NO + O_3_→NO_2_ + O_2_. Ozone depletion potential (ODP) profiles are driven by pesticide production and electricity consumed for irrigation, which in total account for 90 % of ODP impacts as a result of atmospheric emissions (CCl_4_, CBrF_3_, CBrClF_2_) evolved from crude oil production, diesel refinery and natural gas transportation. The DNDC-projected negative NEE, i.e. a net uptake of CO_2_ by the plant–soil ecosystem brings beneficial impacts (negative scores below line) on GWP_100_, which is sufficient to offset environmental burdens (positive scores above line) and leads to an Imola poplar cultivation system with negative C savings at the farm gate.

Although VSRC management consumes less diesel fuel than SRC harvesting (Table 3), generally SRC represents an environmentally advantageous plantation regime over VSRC due to the higher biomass yield and lower irrigation and agrochemical inputs per unit of harvested Imola poplar biomass.

### Cradle-to-biorefinery-gate LCIA profiles for bioethanol production

The main drivers of environmental impacts are cellulase enzyme and chemical inputs, as well as emissions involved in the bioethanol production process (Fig. 5). Imola farming stage (excluding carbon sequestration) accounts for 5–45 % of the environmental burdens of the bioethanol across all impact categories due to the energy and agrochemicals consumed in plantation management and the field emissions released from agricultural land.

Generally enzyme (Cellic Ctec 1) dominates the ‘cradle-to-biorefinery-gate’ environmental profiles of bioethanol, accounting for 50–90 % of burdens (positive scores above the zero line) on abiotic depletion, acidification, GWP_100_ and POCP due to its energy-intensive production process. The emissions evolved from enzyme life cycle (e.g. field emissions from carbon substrate production) contribute to 20–50 % of environmental damage on eutrophication and toxicity impact categories. The surplus electricity co-product of the ethanol production represents an environmental ‘saving’ across all impact categories. This combined with significant GHG ‘savings’ brought by the carbon sequestered in the bioethanol molecules and soil carbon accumulation in the Imola plantation is sufficient to ‘offset’ the positive emissions incurred from the bioethanol production and leads to bioethanol with a net negative GHG balance at the biorefinery gate. The results presented here were conducted on an early variant of the Cellic Ctech production series (Cellic Ctech 1) and advances have been made recently in this series (e.g. Cellic Ctec 3). However, we consider that the level of enzyme required in saccharification stage in this study are modest, likely also for advanced cellulases usage and that the production of advanced cellulase enzymes will remain an important contributor to the cradle-to-gate LCIA profile of 2G bioethanol.

SRC shows environmental advantages over VSRC across almost all impact categories (except for GWP_100_, where comparison is driven by carbon sequestration), explained by the lower irrigation and agrochemical inputs per unit harvested Imola biomass under this regime. The dilute acid (DA) scenario delivers better environmental performances than liquid hot water (LHW) on abiotic depletion, human toxicity impact categories where the higher enzyme loading in LHW is a determining factor for comparison results. However, LHW is environmentally advantageous over DA on GWP_100_, acidification, eutrophication, eco-toxicity and POCP due to the higher surplus electricity production in LHW, additional chemical inputs and induced emissions in DA process, e.g. sulphuric acid input and consequential SO_2_ emissions, ammonia input (for neutralisation) and induced NH_3_ emissions, lime (for flue gas desulphurisation).

### Cumulative cradle-to-grave LCIA profiles of E100 bioethanol vs. petrol

The environmental profiles of Imola poplar-derived E100 bioethanol over its whole life cycle from plantation to use as flex fuel vehicle (FFV) fuel are dominated by the cultivation and bioethanol conversion stages, which are responsible for 70–98 % environmental burdens in total (Fig. 6). Transportation involved in the bioethanol supply chain contributes less than 5 % (Fig. 6). Although GHGs resulted from fuel combustion in the vehicle engine override the negative GWP_100_ score contributed by carbon sequestration into biomass, the environmental benefits brought by soil carbon accumulation and the avoided emission credits from surplus electricity export lead to a final bioethanol product with overall negative GHG balance. Regardless of different pretreatment technologies or plantation management regimes, Fig. 7 shows Imola poplar-derived bioethanol to be overall environmentally superior to petrol in GWP_100_, ODP and POCP impact categories when soil carbon accumulation is taken into account. E100 bioethanol produced from Imola poplar can hardly compete with petrol in acidification, eutrophication, and toxicity impact categories.

The contribution that soil carbon accumulation can make to achieving low-GHG biofuels was a striking finding of the study. The results were therefore further examined to exclude the DNDC-simulated net uptake of CO_2_ by soil. In this case, bioethanol produced from SRC Imola poplar had a GWP_100_ score only some 4 % better than petrol and the VSRC regime had equal GWP_100_ scores (Fig. 7). It is clear that GWP_100_ impacts for Imola poplar-derived bioethanol and the scale of GWP_100_ saving shown for the bioethanol over petrol are very sensitive to the inclusion of soil carbon accumulation projected by DNDC.

To represent general practice in commercial poplar plantation rather than site-specific case in Italy, two alternative irrigation scenarios were modelled in this study—in one scenario fossil fuel is replaced with woody biomass for green electricity generation to meet irrigation supply; in the second scenario flood irrigation without energy requirement is applied in poplar plantation [49]. Generally, switching from national grid supply to woody biomass-generated electricity leads to a 2–10 % improvement in environmental performance of bioethanol, except for terrestrial eco-toxicity where up to 50 % decline in environmental burdens is achieved (Fig. 8). Further reduction in environmental impacts could be achieved if applying flood irrigation in poplar plantation. For E100 bioethanol derived from LHW processing technology demonstrates potentials to move to competitive position regarding petrol in abiotic depletion while adopting a zero-energy-requirement irrigation option.

### Sensitivity analysis of allocation method

An energy-based allocation method is recommended by the EU Renewable Energy Directive [50] as a basis for sharing burdens between co-products and this differs from the allocation by substitution approach adopted as the base-case for this study. We compare these two different allocation approaches in Fig. 9. Sensitivity analyses on allocation approach indicated that the influences of allocation choice on LCIA profiles of bioethanol vary with scenarios modelled and impact categories investigated (Fig. 9). Based on our chosen 10 % sensitivity threshold, cradle-to-grave environmental profiles of bioethanol in GWP_100_, ODP and abiotic depletion are sensitive to the allocation approach. Switching from substitution to energy allocation approach leads to significant increased GWP_100_ scores for E100 bioethanol especially bioethanol derived from SRC Imola (shifted from negative to positive values). Allocation approach was not a sensitive factor in terms of the LCIA comparisons between bioethanol and petrol.

### Sensitivity analysis on characterisation model

As an alternative to the mid-point method CML 2 Baseline 2000, the damage-oriented method Eco-Indicator 99 Hierarchist (EI 99 H) was applied to the LCA model. Results are presented in Fig. 10 and Additional file 1: Tables S16. The results based on EI 99 generally agree with the outcomes based on the CML method in most comparable impact categories except for abiotic depletion, acidification and eutrophication. Unlike the CML method, EI 99 aggregates acidification and eutrophication impacts into a single indicator result. As given in Fig. 10 bioethanol scenarios appear to have lower impacts than petrol over the life cycle in the aggregated acidification/eutrophication EI 99 category; this is different from the CML findings in Fig. 7, where bioethanol incurred higher acidification and eutrophication scores than petrol. This is mainly driven by the different characterisation factors defined in two methods for NH_3_ and NO_x_ which are regarded as important acidifying and eutrophication contributors and given higher weighting factors in EI 99 than CML. Thus EI 99 does not favour petrol life cycles which involve a range of energy-intensive processes from crude oil extraction to refinery operation and evolve NOx and NH_3_ emissions from fossil fuel combustion. In the EI 99 bioethanol DA scenarios significantly higher impacts occurred in mineral resources depletion, but slightly lower burdens on fossil fuel in comparison with petrol (Fig. 10). This finding differs to an extent from the CML results (abiotic depletion in Fig. 7) due to the dominant contribution (over 90 % impacts) in abiotic depletion in CML being from fossil fuel rather than minerals. EI 99 analyses confirm the LCA outcomes based on CML method—Imola-derived bioethanol deliver better environmental performances than petrol on POCP (respiratory organics in EI 99), ODP (ozone layer in EI 99), and GWP100 (climate change in EI 99). Overall, the LCIA comparisons of Imola-derived bioethanol and petrol counterparts are not very sensitive to the characterisation model choice. Similar findings also occur in the LCIA comparisons between different bioethanol scenarios examined under two characterisation methods.

## Conclusions

In order to project impacts caused by land use change (LUC) for perennial bioenergy crop plantation where complex water and nutrient cycling processes incur, the modelling approach adopted should account for entire agro-ecosystem (e.g. atmospheric inputs, soil water dynamics and crop growth) and explicitly represent soil, crop type and land management processes [25]. Generally there are two modelling approaches applicable to estimating field emissions from agricultural lands as a result of LUC—empirical models such as the IPCC Tier 1 methodology [51] and process-oriented models, e.g. DNDC. The former is based on input–output data relations, more suitable for a large-scale or global assessments; whereas, the latter capturing underlying processes and interactions performs better for scenario analyses, e.g. agro-ecosystem change, or projections for new sites or future climate conditions [25, 52]. However, process-based models and especially whole agro-ecosystem modelling approaches are rarely applied in research on perennial bioenergy crops [25]. By adopting the modified DNDC, this study demonstrates the integration of process-based agro-ecosystem modelling approach into broader LCAs of a 2G biofuel and advances the understanding of influences of C/N cycles at perennial bioenergy crop–soil ecosystem on the overall cradle-to-grave environmental profiles of the biofuel under different scenarios. Field emissions from agro-ecosystem projected by the DNDC not only cause environmental burdens on acidification and eutrophication but also bring significantly beneficial effects on POCP and GWP_100_ due to CH_4_ oxidation, NO release (to remove O_3_ via atmospheric reaction) and net uptake of CO_2_ by the plant–soil ecosystem. A particular aspect of the present study that warrants further research attention is the contribution that soil carbon accumulation can make to achieving low-GHG biorefinery products in the future.

The LCA outcomes indicate that significant overall environmental savings are attainable compared with petrol for several Imola poplar bioethanol scenarios. Via the comparative LCA analyses presented here for bioethanol production from SRC/VSRC Imola poplar across potential supply chains the following attributes can be recognised as critical to 2G biofuel profiling. In a sense, these represent priority issues for potential improvement in biofuel profiling based on LCA findings:

- The specific agricultural system applied (VSRC vs. SRC), e.g. advantages from relatively low agrochemical inputs due to high biomass yield, disadvantage of energy-intensive mechanical irrigation system
- Energy source (and demand) for agricultural operation and production system, e.g. energy source for irrigation, potential process energy integration
- Selection of optimal processing technology to lower enzyme loadings for biomass hydrolysis and avoid chemical-intensive pretreatment, but without compromising total outputs from biorefinery
- Importance of profiling methodology for co-products and emissions applied in the LCA study.

Overall, Imola-derived bioethanol represents a promising alternative transport fuel with some environmental savings ranging between 35 and 100 % over petrol in GWP_100_, ODP and POCP. The Imola poplar feedstock for 2G biofuel in Italy is shown in our modelling to offer significant life cycle GWP_100_ savings over petrol when carbon accumulation in the agro-ecosystem is accounted for, placing this biofuel well within the desirable categories being targeted by policymakers internationally (e.g. the EU Renewable Energy Directive [50]). However, Imola-derived bioethanol can hardly compete with petrol in acidification, eutrophication, abiotic depletion, human and eco-toxicity impact categories. Overall environmental sustainability of Imola-bioethanol system could be further improved via process integration and supply chain optimisation to achieve potential trade-off between GHG targets and optimal solutions on broader environmental issues. Such modelling research for optimal design of sustainable Imola-derived biofuel system can be explored in future study.

## Methods

### Plant materials and experimental site

The hybrid poplar clone Imola has very high biomass yield, excellent rooting ability and resistance to rust (by *Melampsora* spp.) leaf disease (by *Marssonina brunnea)* and woolly aphid *Phloeomyzus passerinii* Sign. Imola was grown under two management regimes: short-rotation coppice (SRC, rotation with 5-year harvesting intervals) and very short rotation coppice (VSRC, rotation with 2-year harvesting intervals). The experimental plots for the 2 regimes were of approximately 0.8 ha located at Casale Monferrato (latitude 45°13′N, longitude 8°51′E). The Casale Monferrato region has a sub-continental climate with annual precipitation varying between 600 and 1100 mm and an average mean annual temperature of 13.3 °C (minimum and maximum temperature averaged at 8.2 and 19.7 °C, respectively). The plantation was established in March 2009 at a planting density of 1111 tree/ha and 8333 tress/ha for SRC and VSRC, respectively. Stems were cutback in the following winter to elicit the coppice response which is characterised by the vigorous growth of multiple new stems in the spring. Throughout the Imola growing seasons, N fertilizer, pesticide and herbicide application, mechanical weed control and irrigation were performed and recorded.

### Meteorological and soil profile

Daily meteorological data (temperature, precipitation, wind speed, solar radiation) were collected from an on-site weather station. The soil at Imola plantation in Casale Monferrato is of alluvial origin, formed in the recent sediments of the Po River. The presence of Regosol-type soils can be explained by short pedogenetic evolution, which does not support the development of diagnostic horizons. Two types of soil (classification according to World Reference Base for Soil Resources [53]) are present in the Imola plantation and their fractions differ in SRC and VSRC plots (Table 5). Both soil types belong to Calcaric Regosols classification, but they differ in gravel contents—type A soil (Calcaric, Endoarenic) lacks fragments which are abundant in type B (Calcaric Endskeletic Regosols). Generally, both soil types tend to be alkaline with pH varying between 7.8 and 8.2. The upper 55 cm soil texture is sandy loam (classification according to soil texture triangle [54]) with soil bulk density of 1.3 g/cm^3^, whereas the soil texture at lower level (55/60 to 100 cm) is much course (loamy sand to sand) with the density range of 1.4–1.5 g/cm^3^. Organic carbon content is approximately 0.7 % in topsoil, decreasing significantly with depth. C:N ratio at all soil layers approaches 10. Topsoil (data not shown) is slightly carbonaceous (CaCO_3_ content 5–6 %) and has a low cation exchange capacity (3–9 cmol/kg). Available water content in topsoil and subsoil is about 14 and 3–7 %, respectively. Hydraulic conductivity range (Table 5) was estimated according to Soil Survey Manual [55].

**Table 5:** Soil profiles for Imola plantation at Casale Monferrato

Depth (cm)	Total N (%)	Organic C (%)	C/N	pH (H_2_O)	pH (KCl)	Sand (g kg^−1^)	Silt (g kg^−1^)	Clay (g kg^−1^)	Textural class	Bulk density (g cm^−3)^	Total porosity (% vol)	Field capacity (% vol)	Wilting point (% vol)	Hydro conductivity (cm h^−1)^
Soil type a—Calcaric Regosol (Epiloamic, Endoarenic)^a^														
0–15	0.07	0.69	10.0	7.8	7.3	580	365	55	Sandy loam	1.33	49	16.9	4.7	4.3–5.2
15–34	0.08	0.83	9.8	7.9	7.2	570	385	45	Sandy loam	1.32	50	16.9	4.3	4.3–5.2
34–55	0.05	0.56	10.2	7.8	7.3	540	400	60	Sandy loam	1.33	49	7.6	4.5	4.3–5.2
55–104	0.02	0.25	11.2	8.2	7.6	779	194	27	Loamy sand	1.37	48	9.6	1.7	8.0–29.0
Soil type b—Calcaric Endoskeletic Regosol (Epiloamic, Endoarenic)^a^														
0–18	0.07	0.72	10.6	7.8	7.4	475	475	50	Sandy loam	1.30	50	18.9	4.2	4.2–4.5
18–60	0.07	0.75	10.3	7.9	7.3	510	435	55	Sandy loam	1.27	52	18.7	4.8	4.2–4.5
60–75	0.02	0.22	10.1	8.2	7.7	881	89	30	Sandy	1.38	48	7.4	1.5	10.0–219.0
75–100	0.00	0.06	–	8.2	8.1	982	10	8	Sandy	1.47	44	5.9	0.5	10.0–219.0
Weighted average topsoil profile for SCR^b^														
0–15	0.07	0.71	10.4	7.8	7.37	510	438	52	Sandy loam	1.31	49.67	18.23	4.37	4.23–4.73
Weighted average topsoil profile for VSCR^b^														
0–15	0.07	0.71	10.3	7.8	7.35	528	420	52	Sandy loam	1.32	49.50	17.90	4.45	4.25–4.85

^a^Based on soil classification by World Reference Base for Soil Resources [53]

^b^33.3 % soil a and 66.7 % soil b present in SRC plantation; 50 % soil a and 50 % soil b are present in VSRC plantation

### Field measurements and yield estimation

The height of the poplar stems and the stem diameters at breast height (Dbh, at height of 130 cm) were measured for each successive year since the plantation establishment. The yield of VSRC above-ground woody biomass in the first and third year were determined by destructive harvests; the non-destructive yield of above-ground woody biomass in SRC/VSRC plantations at the end of each non-harvest year were estimated based on the genotype-specific allometric relationship established between stem Dbh, and above-ground biomass weight (measurements obtained from another experimental plantation of *P.* × *canadensis* clones including Imola at the same location). The allometric function development $(Weight_{dry} = 0.0989 \times Dbh^{2.3574} )$ has been addressed in detail by Merlone [56].

### Sampling and processing of VSRC Imola poplar biomass

The stem and branch samples (approx. 20 cm of stem/branch length with bark) with five biological replicates were collected from the re-sprouting shoots (stem samples collected at 130 cm height) at the beginning of VSRC 1st, 2nd, 3rd and SRC 4th growing seasons for enzymatic saccharification and elemental analysis. Leaf and root samples were collected from the VSRC plantation with five biological replicates for elemental analysis.

Prior to saccharification and elemental analysis all stem and branch samples were set out in open trays in the laboratory to equilibrate for 2–3 days at ambient temperature and humidity, further ground individually using a Retsch (M200) cutting mill and sieved to a defined particle size of between 20 and 80 mesh (850–180 µm in one dimension) to achieve a homogenous mixture of biomass [57]. The moisture content of each ground biomass sample was determined by removal of a subsample and oven-drying at 105 °C overnight.

### Saccharification and elemental analysis methods

Saccharification assays were performed according to the NREL protocol [58]. An equal amount of fresh sample equivalent to 0.25 g ODW was incubated with sodium citrate buffer (pH 4.8), 400 µg tetracycline, and 300 µg cyclohexamide. To ensure that enzyme concentration was non-limiting in the assay, the concentration of the cellulase enzyme mix was doubled to approximately 60 FPU/g ODW biomass of cellulase (Celluclast 1.5 L; Novozymes, Bafsvaerd, Demark) and 64 pNPGU/g ODW biomass of β-glucosidase (Novozyme 188; Novozymes, Bafsvaerd, Demark). Distilled water was added to bring the volume of each vial to 10 ml after enzyme addition. A reaction blank was prepared for each sample, containing buffer, water and an identical amount of biomass in 10 ml volume. Samples were incubated for 7 days at 50 °C in a shaking rotary incubator. The glucose concentrations were determined using a HPLC (Agilent Technologies 1200 Series) equipped with a refractive index detector and a BIO-RAD Aminex HPX 87P column at 55 °C, with an elution rate of 0.6 ml/min using H_2_O as mobile phase. Means and standard errors (mean ± SE as a proportion of ODW biomass) were determined from the five biological replicates for glucose release by enzymatic saccharification and for soluble glucose. One-way ANOVA was performed allowing pairwise comparisons between glucose releases from stem and branch material of each age group.

Approximately 1 g of subsamples from stem, branch, leaf and root samples were further ground using Retsch CryoMill (Model 20.748.0001). The elemental analysis for carbon, hydrogen and nitrogen was carried out by OEA Laboratories Ltd using a Thermoquest EA1110 elemental analyser. The results were used to develop C/N partitioning models for DNDC and for the LCA modelling.

### Biogeochemistry model: DNDC

The DNDC model is one of the most well-developed process-oriented biogeochemistry models and has been validated worldwide [59–68]. A relatively complete suite of biogeochemical processes (e.g. plant growth, organic matter decomposition, fermentation, ammonia volatilisation, nitrification, denitrification) has been embedded in the model, enabling computation of transport and transformations in plant–soil ecosystems. DNDC consists of two components: the first component, consisting of the soil climate, crop growth, and decomposition sub-models, converts primary drivers (e.g. climate, soil properties, vegetation, and anthropogenic activity) to soil environmental factors (e.g. temperature, moisture, pH, redox potential, and substrate concentration gradients); the second component, consisting of the nitrification, denitrification, and fermentation sub-models, simulates C and N transformations mediated by the soil microbes.

The soil climate sub-model simulates soil temperature, moisture and redox potential profiles driven by daily weather data in conjunction with soil texture and plant demand for water. The plant growth sub-model calculates crop growth and development driven by air temperature, soil water and nitrogen supplement at daily time step. The decomposition sub-model tracks turnover of soil organic matters that produce CO_2_ emitted from the soil as well as inorganic nitrogen released from mineralisation. The rest three sub-models calculate trace gas emissions from nitrification, denitrification and fermentation, respectively. The six sub-models interacting with each other to describe cycles of water, C and N for the target ecosystem. If any single change in climate, soil or management occurs, the change will simultaneously affect a series of soil environmental factors such as temperature, moisture, redox potential, pH and substrate concentration; and the changes in the soil environmental factors will collectively affect a group of biogeochemical reactions. By integrating the interactions among the primary drivers, the soil environmental factors and the soil biogeochemical reactions, DNDC is capable of predicting impacts of climate change or management alternatives on crop yield and soil biogeochemistry. For example, soil N exists in several pools in DNDC, namely organic N, ammonium, ammonia, and nitrate; whereas, nitrogen dynamics in soil are simulated at an hourly or daily time step through a series of biogeochemical reactions, such as decomposition, microbial assimilation, plant uptake, ammonia volatilisation, ammonium adsorption, nitrification, denitrification and nitrate leaching. The N emissions are predicted as by-products or intermediate products from the relevant N transformation processes, mainly nitrification and denitrification. In DNDC, fertilisation and manure amendment are parameterised to regulate the soil N dynamics in all the N pools. For the application of synthetic fertilizer, DNDC distributes nitrogenous fertilizers into relevant soil N pools based on the application rates and the fertilizer species. For the manure amended in the soil, the N bound in the organic manure is released through decomposition and distributed into relevant soil N pools and then engaged in the soil N cycling in the simulation.

DNDC provides a user-friendly interface to allow for creating new crop species by defining a number of physiological and phenology parameters. Under this study, Imola was introduced as a new crop type in DNDC. The parameters describing the fundamental features of Imola are listed in Table 6, which include maximum annual productions for seeds (minor component, excluded from LCA model), leaves, stem (plus branch) and roots, C/N ratios for the parts of plant, accumulative temperature for maturity, water requirement, accumulative growth temperature, nitrogen fixation index and optimum temperature. However, DNDC was originally developed for agricultural land and crop modelling (particularly annual or perennial food crops, e.g. corn, sugarcane). To meet the demand of the study (modelling 2G bioenergy crops), the plant growth sub-model was modified to enable DNDC to simulate the perennial plants (e.g. poplar) with woody stem (plus branch) or roots. A new input interface was created to allow users to separately define the annual production rates over SRC/VSRC rotations for leaf, stem and root biomass based on their relative proportions and C/N ratio values. Driven by the plant growth routines embedded in DNDC, the daily increment in total poplar biomass is calculated based on the daily temperature, soil water and nitrogen availabilities. The simulated daily increase in biomass is then partitioned into leaf, stem (plus branch), root or seed based on the crop parameters.

**Table 6:** Plant parameters for Imola based on calibration against measured data

Plant parameter	Value	Notes
Maximum seed yield	180	kg C/ha/year
Biomass partitions	0.01/0.16/0.65/0.18	seed/leaf/stem& branch/root
C/N ratio	19/19/426/45	seed/leaf/stem& branch/root
Total N demand	260.5	kg N/ha/year
Thermal degree days (TDD)	3500	°C
Water requirement	100	kg water/kg dry matter
N fixation	1.2	Plant N/N taken from soil
Optimum temperature	18	°C

The simulated senescence allows the leaves to be totally eliminated by the end of the year and a substantial fraction of woody stem (and branch) or root biomass to accumulate into the next year. The annual production of the total tree biomass is constrained with a parabolic curve so that the growth rate decreases along with increase in its age. With the mechanism, the total biomass of stem (and branch) or root biomass could be inter-annually accumulated but with varying rates.

Equipped with the calibrated plant parameters and additional features in DNDC, multi-year dynamics of Imola biomass were simulated for the two management regimes (SRC and VSRC).

### Biorefinery model for Imola poplar bioethanol production

**Fig. 11:**
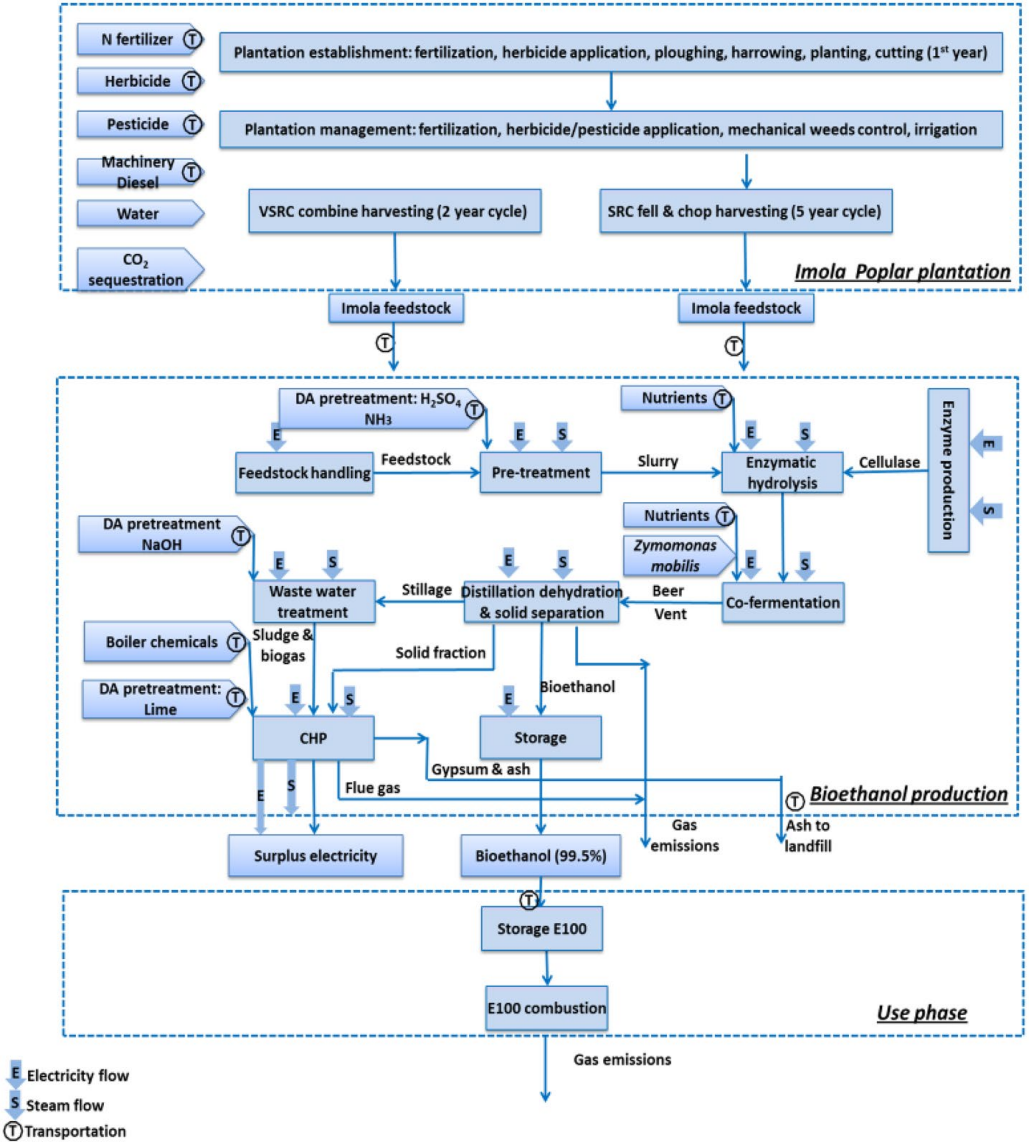
System boundaries for Imola poplar-derived bioethanol

The processes for converting delivered Imola poplar to bioethanol were modelled on a hypothetical biorefinery with a capacity of 2000 oven-dry ton of Imola biomass/day [69]. Two pretreatment technologies (DA and LHW pretreatment) followed by enzymatic hydrolysis, co-fermentation and distillation were modelled (Fig. 11). An energy recovery unit Combined Heat and Power (CHP) and a Wastewater Treatment (WWT) unit were included in the biorefinery model. It was assumed that biorefinery was operated using the electrical and thermal energy recovered from CHP system and the surplus electricity was exported to national grid. The process simulation has been addressed in detailed by Littlewood et al. [70].

### LCA of Imola poplar biofuel case-study

The subsystems modelled within the attributional LCA (aLCA) system boundary for the Imola-based bioethanol were—SRC and VSRC Imola plantation, bioethanol production and biofuel use phase (Fig. 11). The cultivation of SRC and VSRC Imola under this study has been a system with changing land use from marginal land; thus plantation land use change was considered to be a relevant subsystem for this study. Human labour was excluded from the system boundary as is common practice in LCA studies.

The functional unit for the LCA was defined as ‘*100* *km distance driven in a FFV using various fuels compared on an equivalent energy basis*’ to enable the Imola poplar bioethanol to be compared with conventional fossil petrol use.

A ‘substitution’ allocation approach was applied where multiple-products occurred in the bioethanol production stage, i.e. bioethanol plus surplus electrical power generated from the biorefinery’s CHP system and exported from the biorefinery to the Italian national grid. This bio-electricity co-product is assumed to displace the need for that amount of electricity to be generated from fossil fuels within the Italian national grid system. This allocation approach therefore assigns all the environmental burdens of the Imola poplar biomass cultivation and processing to the bioethanol product, but credits the bioethanol with an ‘avoided burdens’ credit of the emissions and fossil fuel consumptions that would have been incurred by generating that amount of electricity conventionally.

A stoichiometric carbon-counting approach was used to ‘track’ the carbon flows from Imola biomass into bioethanol and through into its use as a fuel over the life cycle. This C-counting approach with regard to the bioethanol was applied to determine (1) carbon ‘sequestration’ into the bioethanol (from the Imola poplar cultivation phase of the life cycle, representing a ‘negative’ GHG balance at this stage); (2) soil carbon accumulation in the Imola plantation due to leaf litter and fine root inputs; and (3) releases of carbon during the biorefining processing and the combustion of the bioethanol in the vehicle engine.

A scenario sensitivity analysis method was applied in this study, which involves calculating different scenarios, to analyse the influences of input parameters (soil carbon accumulation) or methodological choices (allocation approach) on either the LCIA output results or rankings [71]. A 10 % change in the characterised LCIA profiles for a single product system or a reversal of the rank order of counterparts in the LCA comparisons were chosen as sensitivity thresholds above which the influence of a parameter or method was considered to be significant for the overall results of the analysis.

The LCA model was implemented using SimaPro^®^ 7.3. A problem-oriented (midpoint) approach—CML 2 baseline 2000 (v2.05) was adopted as the ‘default’ LCIA method; whereas, a damage-oriented approach LCIA method—Eco-Indicator 99 hierarchist version 2.08 defining impact categories at the endpoint level was applied to analyse the sensitivity of the LCA findings to the LCIA methodological choice. Although the impact categories evaluated in two methods seem to differ, most of them overlapped. The CML method represents eco-toxicity in three sub-categories (terrestrial, freshwater and marine aquatic eco-toxicities) whilst Eco-indicators 99 uses one aggregated eco-toxic indicator. Equivalent to POCP in CML method, Eco-indicators 99 includes a respiratory organics category where respiratory impacts as consequences of exposure to organic compounds in summer-smog are evaluated. In addition, Eco-indictors 99 also accounts for winter smog (respiratory effects due to exposure to inorganics), damages induced by radioactive radiation and land conversion and occupation, which are not covered in CML method.

## Additional file

Additional file 1. Supplementary life cycle inventory and impact assessment.

## Abbreviations

2G: second generation
ANOVA: analysis of variance
CHP: combined heat and power
DA: dilute acid pretreatment
Dbh: diameters at breast height
DNDC: denitrification-decomposition
ER: ecosystem respiration
FFV: flex fuel vehicle
GHG: greenhouse gas
GPP: gross primary production
GWP: global warming potential
LCA: life cycle assessment
LCI: life cycle inventory
LCIA: life cycle impact assessment
LHW: liquid hot water pretreatment
LUC: land use change
NEE: net ecosystem exchange
ODP: ozone depletion potential
ODW: oven dry weight
POCP: photochemical oxidation potential
RED: Renewable Energy Directive
SRC: short-rotation coppice
VSRC: very short rotation coppice
WWT: wastewater treatment
