# Supplementary material for: Bioethanol from poplar clone Imola: an environmentally viable alternative to fossil fuel?
Source: Biotechnol Biofuels. 2015 Sep 4;8:134. doi: 10.1186/s13068-015-0318-8 (PMC4558961; doi:10.1186/s13068-015-0318-8)
Supplement: Additional file 1. — Supplementary life cycle inventory and impact assessment. [file 13068_2015_318_MOESM1_ESM.docx]

**Supplementary Information**

**Table S1 Inventory for bioethanol production at the biorefinery per 1 kg ODW poplar processed ^a^**

|  | **DA pretreatment** | **LHW pretreatment** |
| --- | --- | --- |
| **Key parameters** | | |
| Pretreatment ^b^ | 190ºC, 1.1 min,  2.0% sulphuric acid | 200 ºC, 10 min,  water |
| Saccharification ^b^ | Enzyme loading 15 FPU/g glucan  50 ºC, 72 hours | Enzyme loading 15 FPU/g glucan  50ºC, 72 hours |
| Conversion efficiency of glucan to glucose | 86.63% | 56.0% |
| Conversion efficiency of xylan to xylose | 71.78% | 95.83% |
| Fermentation ^c^ | Co-fermentation by recombinant *Zymomonas mobilis,*  32 ºC, 1.5 days  Conversion of glucose and mannose to ethanol 95%,  Conversion of xylose and arabinose to ethanol 85% | |
| WWT ^c, d^ | Biogas composition (dry molar basis) CH_4_ 51% CO_2_ 49%  Total COD removal 99.6% (86% converted to biogas) | |
| CHP ^c^ | Boiler efficiency (feedstock heating value/steam heat) 80% | |
| Flue gas treatment ^c^ | Desulphurisation by adding lime | None |
| **Inputs** | | |
| Poplar (OD kg) | 1.00E+00 | 1.00E+00 |
| Sulphuric acid (93%) (kg) | 2.01E-02 | 0.00E+00 |
| Ammonia (kg) | 7.87E-03 | 0.00E+00 |
| Enzyme Cellic Ctec 1 (kg) | 1.34E-01 | 1.41E-01 |
| Corn steep liquor (kg) | 1.44E-02 | 1.38E-02 |
| Diammonium phosphate (kg) | 1.91E-03 | 1.82E-03 |
| Sorbitol (kg) | 5.79E-05 | 5.47E-05 |
| Caustic (kg) | 6.72E-02 | 0.00E+00 |
| Boiler chemicals (kg) | 5.47E-06 | 4.48E-06 |
| Lime (kg) | 1.77E-03 | 0.00E+00 |
| Cooling tower chemicals (kg) | 6.11E-05 | 6.98E-05 |
| Makeup water ^e^ (kg) | 3.28E+00 | 3.47E+00 |
| **Output** | | |
| Ethanol production (kg) | 2.57E-01 | 2.01E-01 |
| Exported electricity (kWh) | 3.05E-01 | 4.18E-01 |
| Ethanol (kg) | 3.25E-05 | 1.97E-05 |
| CH_4_ (kg) | 1.77E-04 | 2.85E-05 |
| N_2_O(kg) | 5.52E-07 | 5.52E-07 |
| NH_3_(kg) | 7.20E-05 | 0.00E+00 |
| SO_2_(kg) | 1.33E-03 | 5.36E-04 |
| CO(kg) | 3.36E-08 | 3.36E-08 |
| HNO_3_(kg) | 1.14E-05 | 0.00E+00 |
| Landfill disposal of ash (kg) | 2.73E-02 | 2.43E-02 |

##### *a. Derived from reference*[*^1^*](#_ENREF_1)

*b. Based on results reported by Wyman et.al.*[*^2^*](#_ENREF_2)

*c. Based on previous study carried out by National Renewable Energy Laboratory (NREL)* [*^3^*](#_ENREF_3)*.*

*d. WWT includes anaerobic digestion (AD) followed by aerobic treatment. During AD, organic compound (chemical oxygen demand (COD)) removal was assumed as 91% (86% converted to biogas, 5% to cell mass); during aerobic treatment, COD removal was assumed as 99.6% (74% converted to water and CO_2_ 22% to cell mass).*

*e. Water assumed as natural origin.*

**Table S2 Inventory for transport involved in Imola-derived bioethanol supply chains ^d^**

| **Transport** | **Distance** | **Mode** |
| --- | --- | --- |
| On-site transport for VSRC plantation ^a^ | 5.5km | Tractor and trailer |
| On-site transport for SRC plantation ^b^ | 1km | Tractor and trailer |
| Poplar to bioethanol plant | 50km ^c^ | 32-tonne lorry |
| Bioethanol from bio-refinery plant to storage | 160 km ^d^ | 32-tonne lorry |
| Bioethanol from storage to forecourt | 160 km ^d^ | 32-tonne lorry |

1. *Tractor assumed to drive alongside the harvester to collect harvested chips; the transport distance was estimated for a field with row spacing of 3m as 5.5km; during transportation it was assumed a linear loading-weight increase from empty to full capacity*
2. *The transport distance was assumed as 1 km from field to gate; loaded with a full capacity*
3. *Default value for transport from field to bioethanol plant derived from farmed wood was given by the Department for Transport* [*^4^*](#_ENREF_4)
4. *Derived from reference*[*^5^*](#_ENREF_5)

**Table S3 Characterized LCIA profiles of Imola Poplar biomass at farm gate per kg ODW Imola Poplar biomass (method: CML 2 baseline 2000)**

| **Impact category** | **SRC** | **VSRC** |
| --- | --- | --- |
| Abiotic depletion  (kg Sb eq) | 4.57E-01 | 5.36E-01 |
| Acidification(kg SO_2_ eq) | 5.00E-01 | 5.91E-01 |
| Eutrophication(kg PO_4_^3-^ eq) | 1.59E-01 | 1.86E-01 |
| GWP100 (kg CO_2_ eq) | -5.77E+02 | -8.58E+02 |
| ODP (kg CFC-11 eq) | 1.18E-05 | 2.76E-05 |
| Human toxicity(kg 1,4-DB eq) | 2.08E+01 | 3.13E+01 |
| Fresh water aquatic eco-toxicity (kg 1,4-DB eq) | 7.85E+00 | 1.04E+01 |
| Marine aquatic eco-toxicity (kg 1,4-DB eq) | 1.99E+04 | 2.55E+04 |
| Terrestrial eco-toxicity (kg 1,4-DB eq) | 2.43E-01 | 3.11E-01 |
| POCP (kg C_2_H_4_) | 4.77E-03 | 9.00E-03 |

**Table S4 Characterized LCIA profiles of SRC Imola-derived bioethanol (DA) at bio-refinery gate per kg bioethanol (method: CML 2 baseline 2000)**

| **Impact category** | **Total** | **Plantation** | **Pre-treatment** | **Saccharification** | **Fermentation** | **Combustion** | **Make up water** | **Flue gas** | **Surplus electricity** | **Transport** | **C sequestration in poplar feedstock** | **Waste water treatment** | **C sequestration in soil** |
| --- | --- | --- | --- | --- | --- | --- | --- | --- | --- | --- | --- | --- | --- |
| Abiotic depletion  (kg Sb eq) | 1.73E-02 | 1.78E-03 | 6.58E-04 | 1.73E-02 | 5.12E-04 | 1.10E-04 | 0 | 0.00E+00 | -5.34E-03 | 1.54E-04 | 0.00E+00 | 2.08E-03 | 0.00E+00 |
| Acidification  (kg SO_2_ eq) | 2.24E-02 | 1.95E-03 | 1.44E-03 | 1.38E-02 | 4.75E-04 | 6.88E-05 | 0 | 6.68E-03 | -3.49E-03 | 6.51E-05 | 0.00E+00 | 1.38E-03 | 0.00E+00 |
| Eutrophication  (kg PO_4_^3-^ eq) | 3.80E-03 | 6.18E-04 | 5.89E-05 | 1.29E-03 | 4.80E-04 | 9.19E-04 | 0 | 1.02E-04 | -6.63E-04 | 1.77E-05 | 0.00E+00 | 9.68E-04 | 0.00E+00 |
| GWP100  (kg CO_2_ eq) | -2.10E+00 | 4.90E-01 | 7.41E-02 | 2.31E+00 | 6.54E-02 | 4.13E-02 | 0 | 1.44E-02 | -7.58E-01 | 2.08E-02 | -1.91E+00 | 2.95E-01 | -2.74E+00 |
| ODP  (kg CFC-11 eq) | 1.38E-07 | 4.58E-08 | 1.04E-08 | 1.12E-07 | 6.38E-09 | 2.18E-09 | 0 | 0.00E+00 | -5.98E-08 | 3.41E-09 | 0.00E+00 | 1.77E-08 | 0.00E+00 |
| Human toxicity  (kg 1,4-DB eq) | 1.64E+00 | 8.09E-02 | 5.45E-02 | 7.10E-01 | 2.26E-02 | 6.97E-01 | 0 | 5.26E-04 | -1.50E-01 | 5.41E-03 | 0.00E+00 | 2.25E-01 | 0.00E+00 |
| Fresh water eco-toxicity  (kg 1,4-DB eq) | 4.66E-01 | 3.06E-02 | 1.21E-02 | 1.36E-01 | 8.85E-03 | 1.92E-01 | 0 | 0.00E+00 | -8.29E-02 | 2.06E-03 | 0.00E+00 | 1.68E-01 | 0.00E+00 |
| Marine aquatic eco-toxicity  (kg 1,4-DB eq) | 9.25E+02 | 7.73E+01 | 3.81E+01 | 4.64E+02 | 2.25E+01 | 1.69E+02 | 0 | 0.00E+00 | -2.08E+02 | 4.30E+00 | 0.00E+00 | 3.57E+02 | 0.00E+00 |
| Terrestrial eco-toxicity  (kg 1,4-DB eq) | 8.22E-03 | 9.45E-04 | 8.06E-04 | 1.79E-03 | 1.59E-04 | 4.54E-04 | 0 | 0.00E+00 | -1.73E-03 | 4.71E-05 | 0.00E+00 | 5.75E-03 | 0.00E+00 |
| POCP  (kg C_2_H_4_) | 9.83E-04 | 1.85E-05 | 5.97E-05 | 6.61E-04 | 2.05E-05 | 3.33E-06 | 0 | 3.04E-04 | -1.43E-04 | 2.72E-06 | 0.00E+00 | 5.61E-05 | 0.00E+00 |

**Table S5 Characterized LCIA profiles of SRC Imola-derived bioethanol (LHW) at bio-refinery gate per kg bioethanol (method: CML 2 baseline 2000)**

| **Impact category** | **Total** | **Plantation** | **Pretreatment** | **Saccharification** | **Fermentation** | **Combustion** | **Make up water** | **Flue gas** | **Surplus electricity** | **Transport** | **C sequestration in poplar feedstock** | **Waste water treatment** | **C sequestration in soil** |
| --- | --- | --- | --- | --- | --- | --- | --- | --- | --- | --- | --- | --- | --- |
| Abiotic depletion  (kg Sb eq) | 1.71E-02 | 2.28E-03 | 0 | 2.33E-02 | 6.26E-04 | 1.13E-04 | 0 | 0.00E+00 | -9.38E-03 | 1.97E-04 | 0.00E+00 | 0 | 0.00E+00 |
| Acidification  (kg SO_2_ eq) | 1.89E-02 | 2.49E-03 | 0 | 1.86E-02 | 5.81E-04 | 7.43E-05 | 0 | 3.20E-03 | -6.12E-03 | 8.34E-05 | 0.00E+00 | 0 | 0.00E+00 |
| Eutrophication  (kg PO_4_^3-^ eq) | 3.03E-03 | 7.92E-04 | 0 | 1.74E-03 | 5.87E-04 | 1.05E-03 | 0 | 0.00E+00 | -1.16E-03 | 2.26E-05 | 0.00E+00 | 0 | 0.00E+00 |
| GWP100  (kg CO_2_ eq) | -2.87E+00 | 6.28E-01 | 0 | 3.10E+00 | 8.01E-02 | 4.12E-02 | 0 | 3.65E-03 | -1.33E+00 | 2.67E-02 | -1.91E+00 | 0 | -3.50E+00 |
| ODP  (kg CFC-11 eq) | 1.19E-07 | 5.87E-08 | 0 | 1.51E-07 | 7.80E-09 | 2.10E-09 | 0 | 0.00E+00 | -1.05E-07 | 4.37E-09 | 0.00E+00 | 0 | 0.00E+00 |
| Human toxicity  (kg 1,4-DB eq) | 1.62E+00 | 1.04E-01 | 0 | 9.53E-01 | 2.77E-02 | 7.96E-01 | 0 | 2.56E-04 | -2.64E-01 | 6.92E-03 | 0.00E+00 | 0 | 0.00E+00 |
| Fresh water eco-toxicity  (kg 1,4-DB eq) | 3.08E-01 | 3.91E-02 | 0 | 1.82E-01 | 1.08E-02 | 2.19E-01 | 0 | 0.00E+00 | -1.46E-01 | 2.63E-03 | 0.00E+00 | 0 | 0.00E+00 |
| Marine aquatic eco-toxicity  (kg 1,4-DB eq) | 5.83E+02 | 9.90E+01 | 0 | 6.23E+02 | 2.75E+01 | 1.93E+02 | 0 | 0.00E+00 | -3.64E+02 | 5.51E+00 | 0.00E+00 | 0 | 0.00E+00 |
| Terrestrial eco-toxicity  (kg 1,4-DB eq) | 1.34E-03 | 1.21E-03 | 0 | 2.40E-03 | 1.94E-04 | 5.17E-04 | 0 | 0.00E+00 | -3.04E-03 | 6.03E-05 | 0.00E+00 | 0 | 0.00E+00 |
| POCP  (kg C_2_H_4_) | 8.60E-04 | 2.38E-05 | 0 | 8.88E-04 | 2.51E-05 | 2.83E-06 | 0 | 1.68E-04 | -2.50E-04 | 3.49E-06 | 0.00E+00 | 0 | 0.00E+00 |

**Table S6 Characterized LCIA profiles of VSRC Imola-derived bioethanol (DA) at bio-refinery gate per kg bioethanol (method: CML 2 baseline 2000)**

| **Impact category** | **Total** | **Plantation** | **Pretreatment** | **Saccharification** | **Fermentation** | **Combustion** | **Make up water** | **Flue gas** | **Surplus electricity** | **Transport** | **C sequestration in poplar feedstock** | **Waste water treatment** | **C sequestration in soil** |
| --- | --- | --- | --- | --- | --- | --- | --- | --- | --- | --- | --- | --- | --- |
| Abiotic depletion  (kg Sb eq) | 1.76E-02 | 2.08E-03 | 6.58E-04 | 1.73E-02 | 5.12E-04 | 1.10E-04 | 0 | 0.00E+00 | -5.34E-03 | 1.54E-04 | 0.00E+00 | 2.08E-03 | 0.00E+00 |
| Acidification  (kg SO_2_ eq) | 2.27E-02 | 2.30E-03 | 1.44E-03 | 1.38E-02 | 4.75E-04 | 6.88E-05 | 0 | 6.68E-03 | -3.49E-03 | 6.51E-05 | 0.00E+00 | 1.38E-03 | 0.00E+00 |
| Eutrophication  (kg PO_4_^3-^ eq) | 3.90E-03 | 7.23E-04 | 5.89E-05 | 1.29E-03 | 4.80E-04 | 9.19E-04 | 0 | 1.02E-04 | -6.63E-04 | 1.77E-05 | 0.00E+00 | 9.68E-04 | 0.00E+00 |
| GWP100  (kg CO_2_ eq) | -3.19E+00 | 5.54E-01 | 7.41E-02 | 2.31E+00 | 6.54E-02 | 4.13E-02 | 0 | 1.44E-02 | -7.58E-01 | 2.08E-02 | -1.91E+00 | 2.95E-01 | -3.89E+00 |
| ODP  (kg CFC-11 eq) | 2.00E-07 | 1.07E-07 | 1.04E-08 | 1.12E-07 | 6.38E-09 | 2.18E-09 | 0 | 0.00E+00 | -5.98E-08 | 3.41E-09 | 0.00E+00 | 1.77E-08 | 0.00E+00 |
| Human toxicity  (kg 1,4-DB eq) | 1.69E+00 | 1.22E-01 | 5.45E-02 | 7.10E-01 | 2.26E-02 | 6.97E-01 | 0 | 5.26E-04 | -1.50E-01 | 5.41E-03 | 0.00E+00 | 2.25E-01 | 0.00E+00 |
| Fresh water eco-toxicity  (kg 1,4-DB eq) | 4.76E-01 | 4.04E-02 | 1.21E-02 | 1.36E-01 | 8.85E-03 | 1.92E-01 | 0 | 0.00E+00 | -8.29E-02 | 2.06E-03 | 0.00E+00 | 1.68E-01 | 0.00E+00 |
| Marine aquatic eco-toxicity  (kg 1,4-DB eq) | 9.47E+02 | 9.93E+01 | 3.81E+01 | 4.64E+02 | 2.25E+01 | 1.69E+02 | 0 | 0.00E+00 | -2.08E+02 | 4.30E+00 | 0.00E+00 | 3.57E+02 | 0.00E+00 |
| Terrestrial eco-toxicity  (kg 1,4-DB eq) | 8.48E-03 | 1.21E-03 | 8.06E-04 | 1.79E-03 | 1.59E-04 | 4.54E-04 | 0 | 0.00E+00 | -1.73E-03 | 4.71E-05 | 0.00E+00 | 5.75E-03 | 0.00E+00 |
| POCP  (kg C_2_H_4_) | 9.99E-04 | 3.50E-05 | 5.97E-05 | 6.61E-04 | 2.05E-05 | 3.33E-06 | 0 | 3.04E-04 | -1.43E-04 | 2.72E-06 | 0.00E+00 | 5.61E-05 | 0.00E+00 |

**Table S7 Characterized LCIA profiles of VSRC Imola-derived bioethanol (LHW) at bio-refinery gate per kg bioethanol (method: CML 2 baseline 2000)**

| **Impact category** | **Total** | **Plantation** | **Pretreatment** | **Saccharification** | **Fermentation** | **Combustion** | **Make up water** | **Flue gas** | **Surplus electricity** | **Transport** | **C sequestration in poplar feedstock** | **Waste water treatment** | **C sequestration in soil** |
| --- | --- | --- | --- | --- | --- | --- | --- | --- | --- | --- | --- | --- | --- |
| Abiotic depletion  (kg Sb eq) | 1.75E-02 | 2.67E-03 | 0 | 2.33E-02 | 6.26E-04 | 1.13E-04 | 0 | 0.00E+00 | -9.38E-03 | 1.97E-04 | 0.00E+00 | 0 | 0.00E+00 |
| Acidification  (kg SO_2_ eq) | 1.93E-02 | 2.94E-03 | 0 | 1.86E-02 | 5.81E-04 | 7.43E-05 | 0 | 3.20E-03 | -6.12E-03 | 8.34E-05 | 0.00E+00 | 0 | 0.00E+00 |
| Eutrophication  (kg PO_4_^3-^ eq) | 3.16E-03 | 9.27E-04 | 0 | 1.74E-03 | 5.87E-04 | 1.05E-03 | 0 | 0.00E+00 | -1.16E-03 | 2.26E-05 | 0.00E+00 | 0 | 0.00E+00 |
| GWP100  (kg CO_2_ eq) | -4.27E+00 | 7.09E-01 | 0 | 3.10E+00 | 8.01E-02 | 4.12E-02 | 0 | 3.65E-03 | -1.33E+00 | 2.67E-02 | -1.91E+00 | 0 | -4.99E+00 |
| ODP  (kg CFC-11 eq) | 1.98E-07 | 1.38E-07 | 0 | 1.51E-07 | 7.80E-09 | 2.10E-09 | 0 | 0.00E+00 | -1.05E-07 | 4.37E-09 | 0.00E+00 | 0 | 0.00E+00 |
| Human toxicity  (kg 1,4-DB eq) | 1.68E+00 | 1.56E-01 | 0 | 9.53E-01 | 2.77E-02 | 7.96E-01 | 0 | 2.56E-04 | -2.64E-01 | 6.92E-03 | 0.00E+00 | 0 | 0.00E+00 |
| Fresh water eco-toxicity  (kg 1,4-DB eq) | 3.21E-01 | 5.18E-02 | 0 | 1.82E-01 | 1.08E-02 | 2.19E-01 | 0 | 0.00E+00 | -1.46E-01 | 2.63E-03 | 0.00E+00 | 0 | 0.00E+00 |
| Marine aquatic eco-toxicity  (kg 1,4-DB eq) | 6.12E+02 | 1.27E+02 | 0 | 6.23E+02 | 2.75E+01 | 1.93E+02 | 0 | 0.00E+00 | -3.64E+02 | 5.51E+00 | 0.00E+00 | 0 | 0.00E+00 |
| Terrestrial eco-toxicity  (kg 1,4-DB eq) | 1.68E-03 | 1.55E-03 | 0 | 2.40E-03 | 1.94E-04 | 5.17E-04 | 0 | 0.00E+00 | -3.04E-03 | 6.03E-05 | 0.00E+00 | 0 | 0.00E+00 |
| POCP  (kg C_2_H_4_) | 8.82E-04 | 4.49E-05 | 0 | 8.88E-04 | 2.51E-05 | 2.83E-06 | 0 | 1.68E-04 | -2.50E-04 | 3.49E-06 | 0.00E+00 | 0 | 0.00E+00 |

**Table S8 Characterized LCIA comparison of SRC Imola-derived bioethanol (DA) over life cycle per functional unit, 100km driven in a FFV (method: CML 2 baseline 2000)**

| **Impact category** | **Total** | **Plantation** | **Bioethanol conversion process** | **Surplus electricity** | **Transport, poplar to biorefinery** | **C sequestration in poplar feedstock** | **C sequestration in soil** | **Transport, bioethanol** | **Use phase** |
| --- | --- | --- | --- | --- | --- | --- | --- | --- | --- |
| Abiotic depletion  (kg Sb eq) | 1.74E-01 | 1.76E-02 | 2.05E-01 | -5.29E-02 | 1.52E-03 | 0.00E+00 | 0.00E+00 | 2.52E-03 | 0.00E+00 |
| Acidification  (kg SO_2_ eq) | 2.23E-01 | 1.93E-02 | 2.36E-01 | -3.45E-02 | 6.44E-04 | 0.00E+00 | 0.00E+00 | 1.07E-03 | 0.00E+00 |
| Eutrophication  (kg PO_4_^3-^ eq) | 3.79E-02 | 6.12E-03 | 3.79E-02 | -6.57E-03 | 1.75E-04 | 0.00E+00 | 0.00E+00 | 2.89E-04 | 0.00E+00 |
| GWP100  (kg CO_2_ eq) | -1.39E+00 | 4.85E+00 | 2.77E+01 | -7.50E+00 | 2.06E-01 | -1.89E+01 | -2.71E+01 | 3.41E-01 | 1.91E+01 |
| ODP  (kg CFC-11 eq) | 1.42E-06 | 4.54E-07 | 1.47E-06 | -5.92E-07 | 3.38E-08 | 0.00E+00 | 0.00E+00 | 5.58E-08 | 0.00E+00 |
| Human toxicity  (kg 1,4-DB eq) | 1.64E+01 | 8.01E-01 | 1.69E+01 | -1.49E+00 | 5.35E-02 | 0.00E+00 | 0.00E+00 | 8.85E-02 | 0.00E+00 |
| Fresh water aquatic eco-toxicity  (kg 1,4-DB eq) | 4.65E+00 | 3.03E-01 | 5.11E+00 | -8.21E-01 | 2.03E-02 | 0.00E+00 | 0.00E+00 | 3.36E-02 | 0.00E+00 |
| Marine aquatic eco-toxicity  (kg 1,4-DB eq) | 9.22E+03 | 7.65E+02 | 1.04E+04 | -2.06E+03 | 4.26E+01 | 0.00E+00 | 0.00E+00 | 7.04E+01 | 0.00E+00 |
| Terrestrial eco-toxicity  (kg 1,4-DB eq) | 8.21E-02 | 9.36E-03 | 8.87E-02 | -1.71E-02 | 4.66E-04 | 0.00E+00 | 0.00E+00 | 7.70E-04 | 0.00E+00 |
| POCP  (kg C_2_H_4_) | 9.80E-03 | 1.84E-04 | 1.09E-02 | -1.41E-03 | 2.70E-05 | 0.00E+00 | 0.00E+00 | 4.46E-05 | 2.87E-05 |

**Table S9 Characterized LCIA comparison of SRC Imola-derived bioethanol (LHW) over life cycle per functional unit, 100km driven in a FFV (method: CML 2 baseline 2000)**

| **Impact category** | **Total** | **Plantation** | **Bioethanol conversion process** | **Surplus electricity** | **Transport, poplar to bio-refinery** | **C sequestration in poplar feedstock** | **C sequestration in soil** | **Transport, bioethanol** | **Use phase** |
| --- | --- | --- | --- | --- | --- | --- | --- | --- | --- |
| Abiotic depletion  (kg Sb eq) | 1.72E-01 | 2.25E-02 | 2.38E-01 | -9.29E-02 | 1.95E-03 | 0.00E+00 | 0.00E+00 | 2.52E-03 | 0.00E+00 |
| Acidification  (kg SO_2_ eq) | 1.88E-01 | 2.47E-02 | 2.22E-01 | -6.06E-02 | 8.25E-04 | 0.00E+00 | 0.00E+00 | 1.07E-03 | 0.00E+00 |
| Eutrophication  (kg PO_4_^3-^ eq) | 3.03E-02 | 7.84E-03 | 3.34E-02 | -1.15E-02 | 2.24E-04 | 0.00E+00 | 0.00E+00 | 2.89E-04 | 0.00E+00 |
| GWP100  (kg CO_2_ eq) | -9.03E+00 | 6.22E+00 | 3.19E+01 | -1.32E+01 | 2.64E-01 | -1.89E+01 | -3.47E+01 | 3.41E-01 | 1.91E+01 |
| ODP  (kg CFC-11 eq) | 1.23E-06 | 5.81E-07 | 1.59E-06 | -1.04E-06 | 4.33E-08 | 0.00E+00 | 0.00E+00 | 5.58E-08 | 0.00E+00 |
| Human toxicity  (kg 1,4-DB eq) | 1.62E+01 | 1.03E+00 | 1.76E+01 | -2.61E+00 | 6.86E-02 | 0.00E+00 | 0.00E+00 | 8.85E-02 | 0.00E+00 |
| Fresh water eco-toxicity  (kg 1,4-DB eq) | 3.08E+00 | 3.88E-01 | 4.08E+00 | -1.44E+00 | 2.61E-02 | 0.00E+00 | 0.00E+00 | 3.36E-02 | 0.00E+00 |
| Marine aquatic eco-toxicity  (kg 1,4-DB eq) | 5.85E+03 | 9.80E+02 | 8.35E+03 | -3.61E+03 | 5.46E+01 | 0.00E+00 | 0.00E+00 | 7.04E+01 | 0.00E+00 |
| Terrestrial eco-toxicity  (kg 1,4-DB eq) | 1.41E-02 | 1.20E-02 | 3.08E-02 | -3.01E-02 | 5.97E-04 | 0.00E+00 | 0.00E+00 | 7.70E-04 | 0.00E+00 |
| POCP  (kg C_2_H_4_) | 8.59E-03 | 2.35E-04 | 1.07E-02 | -2.48E-03 | 3.46E-05 | 0.00E+00 | 0.00E+00 | 4.46E-05 | 2.87E-05 |

**Table S10 Characterized LCIA comparison of VSRC Imola-derived bioethanol (DA) over life cycle per functional unit, 100km driven in a FFV (method: CML 2 baseline 2000)**

| **Impact category** | **Total** | **Plantation** | **Bioethanol conversion process** | **Surplus electricity** | **Transport, poplar to bio-refinery** | **C sequestration in poplar feedstock** | **C sequestration in soil** | **Transport, bioethanol** | **Use phase** |
| --- | --- | --- | --- | --- | --- | --- | --- | --- | --- |
| Abiotic depletion  (kg Sb eq) | 1.77E-01 | 2.06E-02 | 2.05E-01 | -5.29E-02 | 1.52E-03 | 0.00E+00 | 0.00E+00 | 2.52E-03 | 0.00E+00 |
| Acidification  (kg SO_2_ eq) | 2.26E-01 | 2.28E-02 | 2.36E-01 | -3.45E-02 | 6.44E-04 | 0.00E+00 | 0.00E+00 | 1.07E-03 | 0.00E+00 |
| Eutrophication  (kg PO_4_^3-^ eq) | 3.89E-02 | 7.16E-03 | 3.79E-02 | -6.57E-03 | 1.75E-04 | 0.00E+00 | 0.00E+00 | 2.89E-04 | 0.00E+00 |
| GWP100  (kg CO_2_ eq) | -1.22E+01 | 5.48E+00 | 2.77E+01 | -7.50E+00 | 2.06E-01 | -1.89E+01 | -3.85E+01 | 3.41E-01 | 1.91E+01 |
| ODP  (kg CFC-11 eq) | 2.03E-06 | 1.06E-06 | 1.47E-06 | -5.92E-07 | 3.38E-08 | 0.00E+00 | 0.00E+00 | 5.58E-08 | 0.00E+00 |
| Human toxicity  (kg 1,4-DB eq) | 1.68E+01 | 1.21E+00 | 1.69E+01 | -1.49E+00 | 5.35E-02 | 0.00E+00 | 0.00E+00 | 8.85E-02 | 0.00E+00 |
| Fresh water aquatic eco-toxicity  (kg 1,4-DB eq) | 4.75E+00 | 4.00E-01 | 5.11E+00 | -8.21E-01 | 2.03E-02 | 0.00E+00 | 0.00E+00 | 3.36E-02 | 0.00E+00 |
| Marine aquatic eco-toxicity  (kg 1,4-DB eq) | 9.44E+03 | 9.83E+02 | 1.04E+04 | -2.06E+03 | 4.26E+01 | 0.00E+00 | 0.00E+00 | 7.04E+01 | 0.00E+00 |
| Terrestrial eco-toxicity  (kg 1,4-DB eq) | 8.47E-02 | 1.20E-02 | 8.87E-02 | -1.71E-02 | 4.66E-04 | 0.00E+00 | 0.00E+00 | 7.70E-04 | 0.00E+00 |
| POCP  (kg C_2_H_4_) | 9.97E-03 | 3.47E-04 | 1.09E-02 | -1.41E-03 | 2.70E-05 | 0.00E+00 | 0.00E+00 | 4.46E-05 | 2.87E-05 |

**Table S11 Characterized LCIA comparison of VSRC Imola-derived bioethanol (LHW) over life cycle per functional unit, 100km driven in a FFV (method: CML 2 baseline 2000)**

| **Impact category** | **Total** | **Plantation** | **Bioethanol conversion process** | **Surplus electricity** | **Transport, poplar to bio-refinery** | **C sequestration in poplar feedstock** | **C sequestration in soil** | **Transport, bioethanol** | **Use phase** |
| --- | --- | --- | --- | --- | --- | --- | --- | --- | --- |
| Abiotic depletion  (kg Sb eq) | 1.76E-01 | 2.64E-02 | 2.38E-01 | -9.29E-02 | 1.95E-03 | 0.00E+00 | 0.00E+00 | 2.52E-03 | 0.00E+00 |
| Acidification  (kg SO_2_ eq) | 1.92E-01 | 2.92E-02 | 2.22E-01 | -6.06E-02 | 8.25E-04 | 0.00E+00 | 0.00E+00 | 1.07E-03 | 0.00E+00 |
| Eutrophication  (kg PO_4_^3-^ eq) | 3.16E-02 | 9.18E-03 | 3.34E-02 | -1.15E-02 | 2.24E-04 | 0.00E+00 | 0.00E+00 | 2.89E-04 | 0.00E+00 |
| GWP100  (kg CO_2_ eq) | -2.29E+01 | 7.02E+00 | 3.19E+01 | -1.32E+01 | 2.64E-01 | -1.89E+01 | -4.94E+01 | 3.41E-01 | 1.91E+01 |
| ODP  (kg CFC-11 eq) | 2.01E-06 | 1.36E-06 | 1.59E-06 | -1.04E-06 | 4.33E-08 | 0.00E+00 | 0.00E+00 | 5.58E-08 | 0.00E+00 |
| Human toxicity  (kg 1,4-DB eq) | 1.67E+01 | 1.55E+00 | 1.76E+01 | -2.61E+00 | 6.86E-02 | 0.00E+00 | 0.00E+00 | 8.85E-02 | 0.00E+00 |
| Fresh water aquatic eco-toxicity  (kg 1,4-DB eq) | 3.21E+00 | 5.13E-01 | 4.08E+00 | -1.44E+00 | 2.61E-02 | 0.00E+00 | 0.00E+00 | 3.36E-02 | 0.00E+00 |
| Marine aquatic eco-toxicity  (kg 1,4-DB eq) | 6.13E+03 | 1.26E+03 | 8.35E+03 | -3.61E+03 | 5.46E+01 | 0.00E+00 | 0.00E+00 | 7.04E+01 | 0.00E+00 |
| Terrestrial eco-toxicity  (kg 1,4-DB eq) | 1.74E-02 | 1.53E-02 | 3.08E-02 | -3.01E-02 | 5.97E-04 | 0.00E+00 | 0.00E+00 | 7.70E-04 | 0.00E+00 |
| POCP  (kg C_2_H_4_) | 8.80E-03 | 4.44E-04 | 1.07E-02 | -2.48E-03 | 3.46E-05 | 0.00E+00 | 0.00E+00 | 4.46E-05 | 2.87E-05 |

**Table S12 Ccharacterized LCIA comparison of E100 bioethanol (soil carbon sequestration included) vs. petrol per functional unit, 100km driven in a FFV (method: CML 2 baseline 2000)**

| **Impact category** | **SRC, DA** | **SRC, LHW** | **VSRC, DA** | **VSRC, LHW** | **Petrol** |
| --- | --- | --- | --- | --- | --- |
| Abiotic depletion  (kg Sb eq) | 1.74E-01 | 1.72E-01 | 1.77E-01 | 1.76E-01 | 1.64E-01 |
| Acidification  (kg SO_2_ eq) | 2.23E-01 | 1.88E-01 | 2.26E-01 | 1.92E-01 | 1.02E-01 |
| Eutrophication  (kg PO_4_^3-^ eq) | 3.79E-02 | 3.03E-02 | 3.89E-02 | 3.16E-02 | 1.91E-02 |
| GWP100  (kg CO_2_ eq) | -1.39E+00 | -9.03E+00 | -1.22E+01 | -2.29E+01 | 2.63E+01 |
| ODP  (kg CFC-11 eq) | 1.42E-06 | 1.23E-06 | 2.03E-06 | 2.01E-06 | 3.10E-06 |
| Human toxicity  (kg 1,4-DB eq) | 1.64E+01 | 1.62E+01 | 1.68E+01 | 1.67E+01 | 2.87E+00 |
| Fresh water aquatic eco-toxicity (kg 1,4-DB eq) | 4.65E+00 | 3.08E+00 | 4.75E+00 | 3.21E+00 | 6.08E-01 |
| Marine aquatic eco-toxicity  (kg 1,4-DB eq) | 9.22E+03 | 5.85E+03 | 9.44E+03 | 6.13E+03 | 2.59E+03 |
| Terrestrial eco-toxicity  (kg 1,4-DB eq) | 8.21E-02 | 1.41E-02 | 8.47E-02 | 1.74E-02 | 1.72E-02 |
| POCP (kg C_2_H_4_) | 9.80E-03 | 8.59E-03 | 9.97E-03 | 8.80E-03 | 2.99E-02 |

**Table S13 Ccharacterized LCIA comparison ofE100 bioethanol (soil carbon sequestration excluded) vs. petrol per functional unit, 100km driven in a FFV (method: CML 2 baseline 2000)**

| **Impact category** | **SRC, DA** | **SRC, LHW** | **VSRC, DA** | **VSRC, LHW** | **Petrol** |
| --- | --- | --- | --- | --- | --- |
| Abiotic depletion  (kg Sb eq) | 1.74E-01 | 1.72E-01 | 1.77E-01 | 1.76E-01 | 1.64E-01 |
| Acidification  (kg SO_2_ eq) | 2.23E-01 | 1.88E-01 | 2.26E-01 | 1.92E-01 | 1.02E-01 |
| Eutrophication  (kg PO_4_^3-^ eq) | 3.79E-02 | 3.03E-02 | 3.89E-02 | 3.16E-02 | 1.91E-02 |
| GWP100  (kg CO_2_ eq) | 2.57E+01 | 2.57E+01 | 2.63E+01 | 2.65E+01 | 2.63E+01 |
| ODP  (kg CFC-11 eq) | 1.42E-06 | 1.23E-06 | 2.03E-06 | 2.01E-06 | 3.10E-06 |
| Human toxicity  (kg 1,4-DB eq) | 1.64E+01 | 1.62E+01 | 1.68E+01 | 1.67E+01 | 2.87E+00 |
| Fresh water aquatic eco-toxicity (kg 1,4-DB eq) | 4.65E+00 | 3.08E+00 | 4.75E+00 | 3.21E+00 | 6.08E-01 |
| Marine aquatic eco-toxicity  (kg 1,4-DB eq) | 9.22E+03 | 5.85E+03 | 9.44E+03 | 6.13E+03 | 2.59E+03 |
| Terrestrial eco-toxicity  (kg 1,4-DB eq) | 8.21E-02 | 1.41E-02 | 8.47E-02 | 1.74E-02 | 1.72E-02 |
| POCP  (kg C_2_H_4_) | 9.80E-03 | 8.59E-03 | 9.97E-03 | 8.80E-03 | 2.99E-02 |

**Table S14 Characterized cradle-to-grave LCIA comparison of E100 bioethanol from SRC and VSRC Imola Poplar per functional unit, 100km driven in a FFV (method: CML 2 baseline 2000)**

| **Impact category** | **SRC, DA** | **SRC, DA** | **SRC, DA** | **SRC, LHW** | **SRC, LHW** | **SRC, LHW** | **VSRC, DA** | **VSRC,**  **DA** | **VSRC, DA** | **VSRC, LHW** | **VSRC, LHW** | **VSRC, LHW** | **Petrol** |
| --- | --- | --- | --- | --- | --- | --- | --- | --- | --- | --- | --- | --- | --- |
|  | **grid electricity** | **green electricity** | **flood irrigation** | **grid electricity** | **green electricity** | **flood irrigation** | **grid electricity** | **green electricity** | **flood irrigation** | **grid electricity** | **green electricity** | **flood irrigation** |  |
| Abiotic depletion  (kg Sb eq) | 1.74E-01 | 1.65E-01 | 1.64E-01 | 1.72E-01 | 1.61E-01 | 1.60E-01 | 1.77E-01 | 1.66E-01 | 1.65E-01 | 1.76E-01 | 1.62E-01 | 1.61E-01 | 1.64E-01 |
| Acidification  (kg SO_2_ eq) | 2.23E-01 | 2.17E-01 | 2.16E-01 | 1.88E-01 | 1.80E-01 | 1.80E-01 | 2.26E-01 | 2.19E-01 | 2.18E-01 | 1.92E-01 | 1.83E-01 | 1.82E-01 | 1.02E-01 |
| Eutrophication(kg PO_4_^3-^ eq) | 3.79E-02 | 3.66E-02 | 3.65E-02 | 3.03E-02 | 2.87E-02 | 2.85E-02 | 3.89E-02 | 3.74E-02 | 3.72E-02 | 3.16E-02 | 2.97E-02 | 2.94E-02 | 1.91E-02 |
| GWP100  (kg CO_2_ eq) | -1.39E+00 | -1.50E+00 | -2.73E+00 | -9.03E+00 | -9.17E+00 | -1.07E+01 | -1.22E+01 | -1.23E+01 | -1.38E+01 | -2.29E+01 | -2.31E+01 | -2.50E+01 | 2.63E+01 |
| ODP  (kg CFC-11 eq) | 1.42E-06 | 1.33E-06 | 1.32E-06 | 1.23E-06 | 1.11E-06 | 1.10E-06 | 2.03E-06 | 1.92E-06 | 1.91E-06 | 2.01E-06 | 1.86E-06 | 1.85E-06 | 3.10E-06 |
| Human toxicity  (kg 1,4-DB eq) | 1.64E+01 | 1.61E+01 | 1.60E+01 | 1.62E+01 | 1.58E+01 | 1.57E+01 | 1.68E+01 | 1.65E+01 | 1.64E+01 | 1.67E+01 | 1.63E+01 | 1.62E+01 | 2.87E+00 |
| Fresh water eco-toxicity  (kg 1,4-DB eq) | 4.65E+00 | 4.47E+00 | 4.46E+00 | 3.08E+00 | 2.85E+00 | 2.84E+00 | 4.75E+00 | 4.53E+00 | 4.51E+00 | 3.21E+00 | 2.93E+00 | 2.91E+00 | 6.08E-01 |
| Marine aquatic eco-toxicity  (kg 1,4-DB eq) | 9.22E+03 | 8.79E+03 | 8.76E+03 | 5.85E+03 | 5.29E+03 | 5.25E+03 | 9.44E+03 | 8.91E+03 | 8.88E+03 | 6.13E+03 | 5.44E+03 | 5.40E+03 | 2.59E+03 |
| Terrestrial eco-toxicity  (kg 1,4-DB eq) | 8.21E-02 | 7.68E-02 | 7.63E-02 | 1.41E-02 | 7.26E-03 | 6.58E-03 | 8.47E-02 | 7.82E-02 | 7.76E-02 | 1.74E-02 | 9.11E-03 | 8.28E-03 | 1.72E-02 |
| POCP  (kg C_2_H_4_) | 9.80E-03 | 9.56E-03 | 9.54E-03 | 8.59E-03 | 8.29E-03 | 8.25E-03 | 9.97E-03 | 9.67E-03 | 9.64E-03 | 8.80E-03 | 8.43E-03 | 8.39E-03 | 2.99E-02 |

**Table S15 Sensitivity analysis on allocation approach per functional unit, 100km driven in a FFV (method: CML 2 baseline 2000)**

| **Impact category** | **SRC, DA,**  **substitution** | **SRC, DA, energy** | **SRC, LHW, substitution** | **SRC, LHW, energy** | **VSRC, DA, substitution** | **VSRC, DA, energy** | **VSRC, LHW, substitution** | **VSRC, LHW, energy** | **Petrol** |
| --- | --- | --- | --- | --- | --- | --- | --- | --- | --- |
| Abiotic depletion  (kg Sb eq) | 1.74E-01 | 1.96E-01 | 1.72E-01 | 2.07E-01 | 1.77E-01 | 1.98E-01 | 1.76E-01 | 2.11E-01 | 1.64E-01 |
| Acidification  (kg SO_2_ eq) | 2.23E-01 | 2.22E-01 | 1.88E-01 | 1.94E-01 | 2.26E-01 | 2.25E-01 | 1.92E-01 | 1.98E-01 | 1.02E-01 |
| Eutrophication  (kg PO_4_^3-^ eq) | 3.79E-02 | 3.83E-02 | 3.03E-02 | 3.27E-02 | 3.89E-02 | 3.92E-02 | 3.16E-02 | 3.37E-02 | 1.91E-02 |
| GWP100  (kg CO_2_ eq) | -1.39E+00 | 7.95E+00 | -9.03E+00 | 7.48E+00 | -1.22E+01 | -1.38E+00 | -2.29E+01 | -3.35E+00 | 2.63E+01 |
| ODP  (kg CFC-11 eq) | 1.42E-06 | 1.75E-06 | 1.23E-06 | 1.79E-06 | 2.03E-06 | 2.27E-06 | 2.01E-06 | 2.40E-06 | 3.10E-06 |
| Human toxicity  (kg 1,4-DB eq) | 1.64E+01 | 1.54E+01 | 1.62E+01 | 1.47E+01 | 1.68E+01 | 1.58E+01 | 1.67E+01 | 1.51E+01 | 2.87E+00 |
| Fresh water aquatic eco-toxicity  (kg 1,4-DB eq) | 4.65E+00 | 4.72E+00 | 3.08E+00 | 3.54E+00 | 4.75E+00 | 4.80E+00 | 3.21E+00 | 3.64E+00 | 6.08E-01 |
| Marine aquatic eco-toxicity  (kg 1,4-DB eq) | 9.22E+03 | 9.73E+03 | 5.85E+03 | 7.40E+03 | 9.44E+03 | 9.92E+03 | 6.13E+03 | 7.62E+03 | 2.59E+03 |
| Terrestrial eco-toxicity  (kg 1,4-DB eq) | 8.21E-02 | 8.57E-02 | 1.41E-02 | 3.46E-02 | 8.47E-02 | 8.79E-02 | 1.74E-02 | 3.73E-02 | 1.72E-02 |
| POCP  (kg C_2_H_4_) | 9.80E-03 | 9.68E-03 | 8.59E-03 | 8.66E-03 | 9.97E-03 | 9.82E-03 | 8.80E-03 | 8.83E-03 | 2.99E-02 |

**Table S16 Sensitivity analysis on characterization model per functional unit, 100km driven in a FFV (method: Eco-indicator 99 H)**

| **Impact category** | **Unit** | **SRC, DA** | **SRC, LHW** | **VSRC, DA** | **VSRC, LHW** | **Petrol** |
| --- | --- | --- | --- | --- | --- | --- |
| Carcinogens | DALY | 4.85E-06 | 3.67E-06 | 4.94E-06 | 3.79E-06 | 4.34E-07 |
| Resp. organics | DALY | 1.55E-08 | 1.68E-08 | 1.58E-08 | 1.71E-08 | 1.36E-07 |
| Resp. inorganics | DALY | 1.35E-05 | 1.03E-05 | 1.38E-05 | 1.07E-05 | 1.24E-05 |
| Climate change | DALY | -2.46E-07 | -1.83E-06 | -2.51E-06 | -4.74E-06 | 5.52E-06 |
| Radiation | DALY | 6.36E-08 | 2.34E-08 | 6.58E-08 | 2.63E-08 | 1.05E-08 |
| Ozone layer | DALY | 1.50E-09 | 1.30E-09 | 2.14E-09 | 2.12E-09 | 3.26E-09 |
| Eco-toxicity | PAF*m2yr | 3.62E+00 | 2.08E+00 | 3.74E+00 | 2.23E+00 | 9.90E-01 |
| Acidification/ Eutrophication | PDF*m2yr | 4.93E-01 | 4.16E-01 | 5.06E-01 | 4.32E-01 | 6.80E-01 |
| Minerals | MJ surplus | 3.05E-01 | 1.24E-01 | 3.20E-01 | 1.44E-01 | 3.60E-02 |
| Fossil fuels | MJ surplus | 4.59E+01 | 5.10E+01 | 4.65E+01 | 5.18E+01 | 4.94E+01 |

Note: DALY=Disability Adjusted Life Years; PAF=Potentially Affected Fraction; PDF= Potentially Disappeared Fraction.

**References**

1. J. Littlewood, M. Guo, W. Boerjan and R. J. Murphy, *Biotechnology for Biofuels* 2014.

2. C. E. Wyman, B. E. Dale, R. T. Elander, M. Holtzapple, M. R. Ladisch, Y. Y. Lee, C. Mitchinson and J. N. Saddler, *Biotechnol. Prog.*, 2009, **25**, 333-339.

3. D. Humbird, R. Davis, L. Tao, C. Kinchin, D. Hsu, A. Aden, P. Schoen, J. Lukas, B. Olthof, M. Worley, D. Sexton and D. Dudgeon, National Renewable Energy Laboratory, 2011.

4. DepartmentforTranport, ed. D. f. transport, London, Colorado, USA, 2012.

5. M. Guo, J. Littlewood, J. Joyce and R. Murphy, *Green Chemistry*, 2014, **16**, 4680-4695.
